# Bioactive Lignan Glycosides in Stems of Marsh Rosemary (*Rhododendron tomentosum*): Non-Targeted Screening and Identification Using Two-Stage Analytical Strategy

**DOI:** 10.3390/antiox14040447

**Published:** 2025-04-08

**Authors:** Anna V. Faleva, Danil I. Falev, Aleksandra A. Onuchina, Nikolay V. Ulyanovskii, Dmitry S. Kosyakov

**Affiliations:** Laboratory of Natural Compounds Chemistry and Bioanalytics, Core Facility Center “Arktika”, M. V. Lomonosov Northern (Arctic) Federal University, Northern Dvina Emb. 17, 163002 Arkhangelsk, Russia; d.falev@narfu.ru (D.I.F.); a.onuchina@narfu.ru (A.A.O.); n.ulyanovsky@narfu.ru (N.V.U.); d.kosyakov@narfu.ru (D.S.K.)

**Keywords:** *Rhododendron tomentosum*, lignans, NMR, HPLC-HRMS, non-targeted screening

## Abstract

*Rhododendron tomentosum* is a widespread evergreen shrub used in folk medicine due to the high biological activity of its secondary metabolites, including lignans, that has not been sufficiently studied, and overcoming this problem requires advanced analytical techniques. This study proposes a two-stage analytical strategy for non-targeted screening and identification of lignans in plant extracts that involves the detection of specific lignan-related structures by 2D NMR and the establishment of corresponding fragment ions for further mass spectrometry analysis (HPLC-ESI-MS/MS). The polyphenolic fraction of *R. tomentosum* stem extract was the object of the study. Eight secoisolariciresinol-type lignans (xylosides, glucosides, and rhamnoside), including one previously unknown compound (5-methoxysecoisolariciresinol 9-xyloside), were identified for the first time. The structures of the five compounds were additionally confirmed by preparative HPLC isolation and NMR studies. All of the obtained compounds had antioxidant activity (FRAP and DPPH) similar to that of ascorbic acid. The proposed analytical strategy can be considered an efficient tool for rapid and reliable group screening and identification of lignan derivatives in plant extracts. Its application in the study of *R. tomentosum* extracts has revealed a number of lignan glycosides that may contribute to the medicinal properties of the plant.

## 1. Introduction

Marsh rosemary, also known as *Rhododendron tomentosum* or *Ledum palustre* L., is a small, evergreen shrub that is widely distributed throughout Central and Northern Europe, as well as Northern Asia and North America [1]. It is commonly used in traditional folk medicine to treat bacterial and viral infections, digestive disorders, lung, skin, and internal diseases, as well as rheumatic conditions [2,3,4]. Hypouricemic properties of *R. tomentosum* were also noted [5]. Its extracts possess pronounced insecticidal, acaricidal, and/or insect repellent properties [6,7].

In the literature, studies of *R. tomentosum* chemical composition have focused mainly on the characterization of isoprenoid compounds of essential oils. Moreover, oxygenated sesquiterpenes (ledol, palustrol, and cyclocolorenone isomers), bicyclic monoterpenoids (ascaridole and others), and monoterpene hydrocarbons (*p*-cymene, myrcene, and limonene) have also been detected [8,9,10,11,12,13,14,15,16]. Other substances in *R. tomentosum* extracts include pentacyclic triterpenoids (uvaol, uvaol acetate, ursolic acid, ursolic acid acetate, lupeol, α-amyrin, taraxerol, and others) [17,18] and low-molecular-weight polyphenols, most of which are flavonoids (quercetin, hyperoside, catechin, epicatechin, and others) and coumarins (scopoletin, esculetin, fraxetin, fraxidin, and others) [17,19,20].

Many species of the genus Rhododendron (*R. mariae, R. minutiflorum, R. alutaceum,* and others) contain bioactive lignans of various structures in stems and leaves [21]. However, their presence in *R. tomentosum* has not been sufficiently investigated. To date, only one study has reported the detection of lignan medioresinol in the supercritical CO_2_ extract of its leaves and stems [19]. However, the authors used low-resolution HPLC-MS/MS and available literature data for identifying the detected compounds.

Lignans are low-molecular-weight polyphenols of plant origin that contain two phenylpropane units in their structure. They possess a wide range of biologically active properties (hepatoprotective, antioxidant, antitumor, and others), and their glycosylated derivatives are of great interest owing to their increased bioavailability [22].

Currently, gas chromatography (GC) and high-performance liquid chromatography (HPLC), combined with mass spectrometry (MS), are mainly used for the highly sensitive determination of lignans in plant biomass. GC–MS is a less popular technique because of the low volatility of lignans and the need for labor- and time-consuming derivatization procedures [23,24]. HPLC-MS (MS/MS) is characterized by its high sensitivity and selectivity when analyzing lignans and has been successfully employed in several studies. Thus, this technique was implemented in the quantification of lariciresinol, matairesinol, pinoresinol, and secoisolariciresinol in foods [25] and for the determination of a wide variety of lignans in cereals, oilseeds, and nuts [26], including flax seeds [27]. However, most of the available publications on HPLC–MS analysis of plant extracts address the targeted determination of specific lignans, while the non-targeted screening of unknown compounds of this class in extremely complex matrices is challenging and still not fully resolved.

As shown earlier, one of the most promising approaches for the detection and identification of secondary plant metabolites involves the combination of 2D NMR and HPLC-HRMS [28,29,30] since the former allows the specific structural fragments in complex mixtures to be revealed, and the latter provides molecular-level analysis capabilities in the detection of parent compounds. This combination has previously been successfully used to analyze lignans and other substances in the knotwood of various coniferous trees [31].

In this study, we propose to further develop this methodology by applying a novel two-stage analytical strategy based on 2D NMR and HPLC-HRMS for the non-targeted screening and identification of lignans in an extract of *R. tomentosum*. It is based on the idea that NMR data can be used for the prediction of corresponding fragment ions in tandem mass spectra for further high-resolution mass spectrometry analysis, which greatly simplifies HPLC-HRMS data mining and interpretation.

## 2. Materials and Methods

### 2.1. Chemicals

Methylene chloride (for HPLC, Khimmed, Moscow, Russia) and methanol (HPLC grade, Khimmed, Moscow, Russia) were used in plant biomass extraction. HPLC gradient-grade acetonitrile (Cryochrom, St. Petersburg, Russia), ACS reagent-grade (≥96%) formic acid (Sigma–Aldrich, St. Louis, MO, USA), and type I water obtained from a Milli-Q system (Millipore, Molsheim, France) were used in the preparation of mobile-phase preparative HPLC. For the NMR studies, deuterated dimethyl sulfoxide (DMSO-d_6_, ≥99.8%, Merck, Darmstadt, Germany) was used as the solvent. DPPH (2,2-diphenyl-1-picrylhydrazyl radical, Sigma–Aldrich, Steinheim, Germany), TPTZ (2,4,6-tripyridyl-S-triazine, 99%, Sisco Research Laboratories, Mumbai, India), iron(II) sulfate (FeSO_4_, chem. pure, Veton, Moscow, Russia), iron(III) chloride (FeCl_3_, chem. pure, Khimmed, Moscow, Russia), and hydrochloric acid (HCl, chem. pure, Neva-Reaktiv, St. Petersburg, Russia) were used in the antioxidant activity determination procedures. Secoisolariciresinol (98%), isolated from the compression wood of coniferous species [32], and ascorbic acid (puriss. p.a., ≥99.0%), obtained from Sigma–Aldrich (Steinheim, Germany), were used as standards of known antioxidants in the FRAP and DPPH assays.

### 2.2. Plant Material and Preparation of Extract

*R. tomentosum* plant materials were collected in the Primorsky district of the Arkhangelsk region (Russia) in July 2023. Voucher specimen no. NARFU2023-RhT was deposited in the herbarium of the Northern (Arctic) Federal University. Secondary metabolites were extracted with a methanol–dichloromethane mixture (1:1, *v*/*v*) from the finely ground and carefully averaged stems of *R. tomentosum* (10 g dry weight), using a solvent/sample ratio of 10 mL g^−1^ in three 30 min stages under sonication (35 kHz) in an ultrasonic bath (Sapphire, Moscow, Russia). The obtained extract was fractionated by column chromatography using a Polygoprep 60–50 C18 octadecyl silica column (Macherey-Nagel, Duren, Germany) and four different solvents in order of decreasing polarity [33]. As a result, four fractions of the extract were obtained: E1 (water), E2 (aqueous methanol mixture, 1:1, *v*/*v*), E3 (methanol), and E4 (methanol–dichloromethane mixture, 1:1, *v*/*v*). The full separation procedure was presented in detail in previous work [30]. The total extract yield was 5% (calculated based on dry matter), with 1% being the aqueous methanol extract (E2) with the highest total polyphenol content (325 ± 17 mg eq. GA/g) among the four extracts obtained.

### 2.3. NMR Spectroscopy

One-dimensional ^1^H and two-dimensional (2D) ^1^H-^13^C HSQC, ^1^H-^13^C HMBC, and ^1^H-^1^H TOCSY NMR spectra of the polyphenolic fraction (F2) or isolated individual compounds were acquired in DMSO-d_6_ at 298 K on an AVANCE III 600 spectrometer (Bruker, Ettlingen, Germany) with an operating frequency for protons of 600 MHz. Pulse sequences from the standard Bruker library were implemented. Topspin 3.2 software from Bruker (Ettlingen, Germany) was used to acquire and process the experimental data. The cross-peak assignment for identifying specific lignan structures was performed using ACD/Structure Elucidator expert system software version 2019 from ACD/Labs (Toronto, ON, Canada). Examples of 2D NMR lignan compositional analyses are presented in Figures S3 and S4 (Supplementary Materials).

### 2.4. Liquid Chromatography–High-Resolution Mass Spectrometry (LC-HRMS)

Analysis of the extracted samples was performed using LC-HRMS. The analysis was carried out using an HPLC-HRMS system, which consisted of an LC-30 liquid chromatograph from Shimadzu (Kyoto, Japan) equipped with a UV–Vis diode array spectrophotometric detector and an Orbitrap ID-X high-resolution mass spectrometer from Thermo Scientific (Waltham, MA, USA), which included linear and orbital ion trap mass analyzers. An OptaMax NG ion source for the mass spectrometer was equipped with a heated electrospray ionization (HESI) probe. The conditions of chromatographic separation and mass spectrometric detection were presented in detail in previous work [30]. The identification procedure involved a targeted search for specific product ions in MS/MS chromatograms (dd-MS^2^) corresponding to the fragments discovered by 2D NMR. The detected signals were attributed to the peaks in the total ion current (TIC) MS^1^ chromatogram; thus, the deprotonated molecules of the parent metabolites belonging to the target family were found. Their identification was carried out via accurate mass-based elemental compositions, MS/MS fragmentation patterns, and on-line library searches (Pubchem, Lotus, and Chemspider).

### 2.5. Preparative Chromatography and Fraction Purity Assessment

Preparative high-performance liquid chromatography separation was carried out on an LC-20 Prominence preparative HPLC system from Shimadzu (Kyoto, Japan). The components of the chromatograph, semi-preparative column, mobile phase composition, and column temperature were described in previous work [29]. The mobile phase flow rate was 22.0 mL/min, and the detection wavelength was 280 nm. The gradient was programmed as follows: 0 min—20% B; 15 min—20% B; 17.5 min—100% B, held for 7.5 min. The total separation time was 25 min.

The extract (30.0 mg) was dissolved in 2 mL of aqueous methanol (50%) and injected to the semi-preparative column. The fractions corresponding to the main chromatographic peaks (Figure S5) were collected in glass vials and then evaporated under vacuum in a rotary evaporator until dryness. The purity of the obtained fractions was assessed via additional chromatographic analysis on the same HPLC system using a Nucleodur C18 Gravity analytical column from Macherey-Nagel (Duren, Germany), 5 μm, 250 × 4.6 mm. The percentage of the target compound was determined by comparing the peak area of the target compound in the chromatogram with the total area of all peaks present in the chromatogram.

The characteristics of the obtained fractions (F1–F4) were as follows. F1: light beige powder; yield: 3.3 (0.37)% from extract (raw materials (RM)); purity 93%; UV (MeOH): λ max 228, 280 nm. F2: light beige powder; yield: 2.6 (0.21)% from extract (RM); purity 93%; UV (MeOH): λ max 228, 280 nm. F3: light beige powder; yield: 0.24 (0.02)% from extract (RM); purity 98%; UV (MeOH): λ max 228, 280 nm. F4 (two compounds): light beige powder; yield: 0.39 (0.032)% from extract (RM); purity 98%; UV (MeOH): λ max 228, 280 nm. The ^1^H NMR (600 MHz, DMSO-d_6_) and ^13^C NMR (150 MHz, DMSO-d_6_) data for all of the obtained fractions are presented in Tables S1 and S2.

### 2.6. In Vitro Analysis of Antioxidant Activity

The antioxidant potential was determined via two commonly used methods: the radical scavenging assay (DPPH) and the ferric reducing antioxidant power (FRAP) assay. The FRAP assay was performed according to the method of Benzie and Strain [34] with minor modifications. The FRAP reagent was prepared fresh daily from acetate buffer with pH 3.6, 10 mM TPTZ solution in 40 mM HCl, and 20 mM FeCl_3_ × 6H_2_O in relative proportions of 10:1:1 (*v*/*v*), respectively, and was warmed to 37 °C prior to use. Next, 10 µL of the sample with a concentration of 50 mg/L was mixed with 90 µL of water and 200 µL of FRAP reagent. The absorbance was determined at 593 nm in a 96-well plate on a FlexA-200HT Microplate Reader from Hangzhou Allsheng Instruments Co. (Hangzhou, China) after incubation at 37 °C for 10 min. The results are expressed in mmol Fe^2+^ per gram of sample. The calibration was carried out immediately before the analysis using FeSO_4_ × 7H_2_O solutions in water (0–20 µM).

The DPPH assay was carried out according to the known procedure [35] with minor modifications. The DPPH reagent (0.3 mM) was freshly prepared daily, and accurately weighed samples (~6 mg) were dissolved in 50 mL of ethanol and incubated for 1 h in the dark prior to use. Next, 50 µL of the sample with a concentration of 20–100 mg/L was mixed with ethanol (50 µL) and 0.3 mM DPPH reagent (100 µL). The absorbance was measured at 517 nm after incubation at room temperature for 30 min in the same way as in FRAP assay. Antioxidant activity (IC_50_) was calculated from three parallel measurements.

All of the assays were performed in triplicate. The results of the parallel measurements were analyzed using the descriptive statistics tool of OriginPro 2019b software (OriginLab Corp., Northampton, MA, USA) at a significance level of *p* < 0.05. Data are expressed as mean ± standard deviation.

## 3. Results and Discussion

### 3.1. Targeted Screening of Lignans by 2D NMR (Stage 1)

The 2D HSQC NMR spectrum of the polyphenolic fraction (E2) is shown in Figure S1. For the targeted search for lignans, the region of δC/δH 30–100/1.5–5.0 ppm, in which the main signals characteristic of lignans are concentrated [31,32], was studied. The combination of HSQC data with correlations from the HMBC and TOCSY spectra made it possible to establish the presence of two types of lignans (Figure S2) possessing aryltetralin and dibenzylbutane structures with 9(9′) oxygen atoms [36,37]. The most intense cross-peaks, observed at δC/δH 105.6/6.35, 106.9/.6.56, 40.7/4.26, 44.3/1.91, and 38.5/1.50 ppm, were assigned to 5,5′-dimethoxyisolariciresinol, a representative of the aryltetralin family (Figure S3). The second group of signals (δC/δH 106.0/6.35, 39.4/2.02, 42.1/1.84, and 33.9/2.57 ppm) indicated the presence of secoisolariciresinol structures, related to dibenzylbutane-type lignans (Figure S4). However, the correlations observed in the HMBC spectrum (δC/δH 106/6.35 ppm) revealed that the aromatic component of the identified structures possessed two methoxyl groups as substituents in the 3- and 5-positions of the benzene ring. At the same time, the presence of lignans with one aromatic methoxyl group could not be excluded. In addition, the observed correlations supported the conclusion that the aliphatic hydroxyl groups at position 9 (or 9′) were bonded with a carbohydrate fragment (H(Glu) → C(9) 68.6/4.11 ppm).

### 3.2. Screening of Lignans by HPLC-ESI-HRMS (Stage 2)

**Figure 1:**
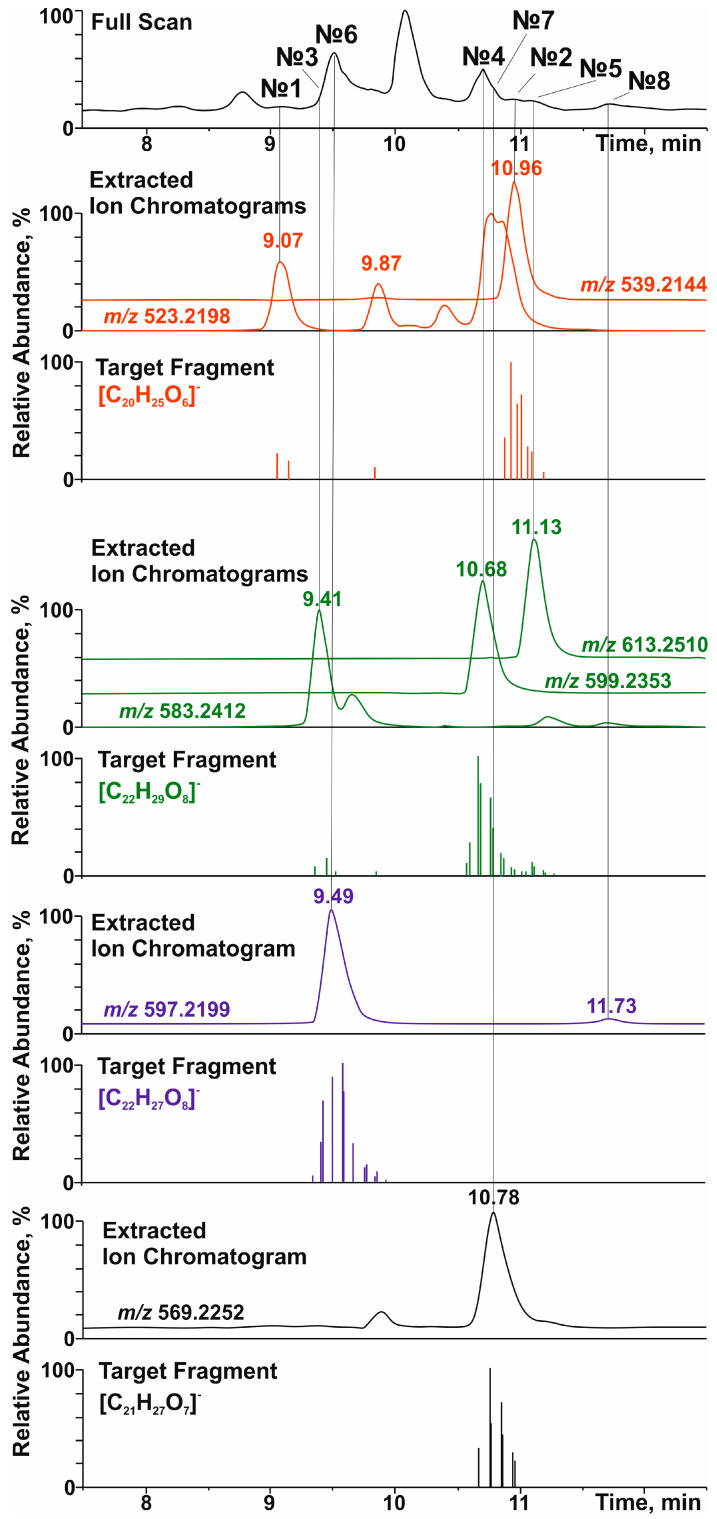
Total ion current (full scan), accurate mass extracted, and selected product-ion (target fragment) chromatograms of lignans in the polyphenolic fraction of the extract of *R. tomentosum* stems.

**Figure 2:**
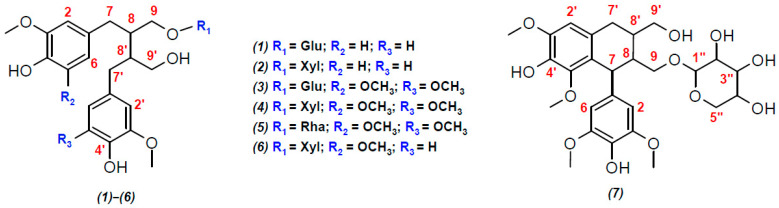
Structural formulas of lignans, presumably identified in the polyphenolic fraction of the extract from the stems of *R. tomentosum* by HPLC-ESI(–)-MS/MS: (**1**) secoisolariciresinol glucoside; (**2**) secoisolariciresinol xyloside; (**3**) 5,5′-dimethoxy-secoisolariciresinol glucoside; (**4**) ssioriside; (**5**) 5,5′-dimethoxy-secoisolariciresinol rhamnoside; (**6**) methoxysecoisolariciresinol xyloside; and (**7**) lyoniside. Explanation for different colors: red—the atom numbers; blue—substituents.

The group screening of lignans in polyphenolic fraction E2 by HPLC-ESI-HRMS was carried out by searching for specific product ions in the tandem mass spectra. The latter were selected on the basis of the 2D NMR data and represented four aryltetralin and dibenzylbutane aglycone structures bearing one or two aromatic methoxyl groups (Table 1, Figure 1). This allowed for the detection of at least eight glycosylated lignans belonging mainly to glucosides and xylosides (Table 1). The structural formulas of all of the tentatively identified (on the basis of accurate mass and tandem mass spectra) compounds are shown in Figure 2.

Among aryltetralin-type lignans, only one compound with a retention time (tR) of 9.5 min was identified. The observation of a signal at *m*/*z* 419.1726 ([C_22_H_27_O_8_]^−^) in its tandem mass spectrum (Figure 1 and Figure S6) indicated the presence of 5,5′-dimethoxyisolariciresinol structural fragments. In an ion source, this metabolite formed an adduct with formic acid ([M-H+FA]^−^) with *m*/*z* 597.2199. This was confirmed by the cleavage of a neutral fragment with a mass of 46 Da (HCOOH) during collisionally activated dissociation (Figure S6). The loss of another neutral fragment with a mass of 132 Da (C_5_H_8_O_4_) indicated that 5,5′-dimethoxyisolariciresinol was present in *R. tomentosum* as a pentose conjugate derivative, presumably lyoniside (**7**, Table 1).

Importantly, in addition to lyoniside, another compound (**8**) with the same elemental composition and retention time of 11.7 min was detected in the extracted ion (*m*/*z* 597.2199, [M-H+FA]^−^) chromatogram (Figure 1). Despite the similar loss of formic acid and pentose moiety (Figure S7), the fragmentation of the aglycone structure differed significantly from that of 5,5′-dimethoxyisolariciresinol. In addition, the different chromatographic retention time indicated clear differences in the structure of the aglycone (other than the aryltetraline-type lignan). However, it was not possible to accurately determine its structure by HRMS.

Dibenzylbutanes with 9(9′) oxygen atoms were the second type of lignans, the group search for which was carried out on the basis of characteristic fragments in the tandem mass spectra. In this case, the product ions with *m*/*z* 361.1666 ([C_20_H_25_O_6_]^−^), 391.1774 ([C_21_H_27_O_7_]^−^), and 421.1880 ([C_22_H_29_O_8_]^−^) were used in the screening procedure. They corresponded to the deprotonated molecules of secoisolariciresinol, methoxysecoisolariciresinol, and dimethoxysecoisolariciresinol, respectively. Two detected compounds with retention times of 9.07 and 10.96 min and product ion [C_20_H_25_O_6_]^−^ were present in the studied polyphenolic fraction (Figure 1). The first chromatographic peak was attributed to the parent analyte with *m*/*z* 523.2198 ([M-H]^−^) and elemental composition C_26_H_36_O_11_ (Δ*m*/*z* = 2.3 ppm). Its tandem mass spectrum (Figure S8) showed the elimination of a hexose residue (162 Da), which allowed for the identification of this compound as secoisolariciresinol glucoside (**1**) with a high degree of confidence. The second compound (tR = 10.96 min) formed an adduct with formic acid ([M-H+HCOOH]^−^) under ESI(–) conditions, which led to the appearance of a signal at *m*/*z* 539.2144 (C_25_H_34_O_10_, Δ*m*/*z* = 1.89 ppm). Its tandem mass spectrum (Figure S9) showed the loss of two neutral fragments corresponding to formic acid (46 Da) and a pentose residue (132 Da), as well as a set of fragment ions that were similar to those of secoisolariciresinol glucoside. On this basis, this lignan was preferably identified as secoisolariciresinol xyloside (**2**).

The search for the characteristic fragment ion [C_21_H_27_O_7_]^−^ revealed only one representative monomethoxy derivative of secoisolariciresinol with tR = 10.78 min. According to the MS and MS/MS spectra (Figure 1 and Figure S10), this compound also formed an adduct with formic acid in the ion source and lost two neutral fragments (46 Da and 132 Da) during collisionally activated dissociation. On this basis, this metabolite was identified as methoxysecoisolariciresinol xyloside (**6**).

The greatest diversity was found in lignan derivatives, the aglycone part of which was represented by 5,5-dimethoxysecoisolariciresinol. In total, 3 metabolites (tR = 9.41, 10.68, and 11.13 min) giving the product ion [C_21_H_27_O_7_]^−^ were detected (Figure 1). Only the least retained analyte did not form an adduct with formic acid in the ion source and lost a neutral fragment with a mass of 162 Da in the collision cell (Figure S11). In addition to the sugar fragment (132 Da), the other two metabolites exhibited cleavage of formic acid (Figures S12 and S13). Although the tandem mass spectra of the detected lignans had a similar set of product ions, the described fragmentation patterns and elemental compositions made it possible to identify them as 5,5-dimethoxysecoisolariciresinol glucoside (**3**), xyloside (**4**), and rhamnoside (**5**), in the order of their elution.

All of the proposed structures were consistent with the results of the NMR analysis and confirmed the presence of dibenzylbutane- and aryltetraline-type lignans in the polyphenolic fraction of the plant extract. A literature search revealed that lyoniside and ssioriside were previously found in the stem extracts of *Vaccínium myrtíllus* [38] and *Rhododendron mucronulatum* [39]. Thus, they can be considered characteristic lignans for representatives of the family Ericaceae.

Thus, the analytical strategy based on 2D NMR and HPLC-HRMS was proven to be promising for the non-targeted screening and identification of lignans and can be applied to other plant extracts for which the major components belong to the same class of compounds. However, this approach is not suitable for the screening and identification of trace amounts of metabolites due to the limited sensitivity of NMR spectroscopy, particularly for complex mixtures such as plant extracts.

### 3.3. Isolation and Characterization of Individual Lignans

To obtain pure preparations of the individual lignans detected by NMR and HPLC-HRMS, semi-preparative reversed-phase HPLC was used. The obtained chromatogram of the polyphenolic fraction with the indicated fraction (F1–F4) collection periods, which are presumably related to the elution of lignans, is shown in Figure S5. The achieved chromatographic resolution ensured an acceptable separation of components and their purity (92.7–97.8%), which was suitable for further structural elucidation by NMR spectroscopy techniques. The obtained ^1^H, ^13^C, and 2D NMR data are presented in Tables S1 and S2 and in Figures S14–S27.

From the evidence presented in the ^1^H and 2D NMR spectra, the analytes in fractions F1 and F2 were concluded to be lyoniside (**7**) and ssioriside (**4**). The signals from the protons of the aromatic hydroxyl groups (δH ~ 8.0 ppm) observed in the ^1^H NMR spectra confirmed that an aliphatic hydroxyl was involved in the formation of a simple ether bond with xyloside.

To the best of our knowledge, compound (**6**), constituting fraction F3, is a new lignan xyloside first discovered in plants. Detailed NMR data analysis suggested that its carbon skeleton was similar to that of ssioriside and secoisolariciresinol xyloside. However, the HMBC correlations (Figure S28) of H-2 and H-6 (δH 6.34) with C-3 and C-5 (δC 147.6) atoms indicated that one aromatic ring had a methoxy group not only at position 3 but also at position 5. Moreover, δH 6.34 was correlated with C-7 (δC 33.56), and H-7 (δH 2.54) was correlated with δH 2.01 (H-8) and δH 3.81 and 3.31 (H-9) in the TOCSY spectrum (Figure S23 and S28). Additionally, H-1” of the xyloside moiety (δH 4.06) correlated with C-9 of aglycone (δC 68.7), according to the HMBC data. This confirmed that the aliphatic hydroxyl group from the dimethoxylated structure was involved in the formation of ether bonds. Thus, compound (**6**) was identified as 5-methoxysecoisolariciresinol 9-xyloside.

Fraction F4 contained two lignans. One of them, secoisolariciresinol xyloside (**2**), was successfully identified by HPLC-ESI(–)-HRMS. The second component also possessed the xyloside moiety in its structure, but the carbon skeleton of the aglycone differed significantly from those of the aryltetralin and dibenzylbutane types of lignans. The HMBC spectrum (Figure S26) indicated that both aromatic rings had methoxy groups at positions 3 and 5, but one of them (δH 6.56; H-2′ and H-5′) was correlated with an oxygenated CH_2_ group (δC 81.5; C-7), which was involved in the formation of an ether bond with the oxygen of the propane chain from the second aromatic ring. The main correlations between the propane chains were analyzed by TOCSY NMR (Figure S27 and S28). Thus, this aglycone was identified as lariciresinol and, in conjunction with xyloside (H-1” → C-9; 66.06/4.16 ppm), it formed the well-known plant metabolite prupaside (**8**).

The main signals distinguishing the identified lignans resonated from the atoms in the aliphatic chain at positions 7 (7′), 8 (8′), and 9 (9′). In Tables S1 and S2, the diagnostic NMR signals that were used to distinguish these lignans from each other are highlighted in bold for subsequent studies on the composition of plant extracts using NMR spectroscopy.

### 3.4. Antioxidant Activity

The antioxidant activities of the isolated fractions of individual compounds (F1–F4) and the whole polyphenolic fraction of the extract (E2) were evaluated by two independent methods. These included (i) detecting the ability of an antioxidant to neutralize free radicals by donating hydrogen atoms (DPPH), and (ii) determining the ability of an antioxidant to reduce any compound [40] through electron transfer. Standard samples of secoisolariciresinol and ascorbic acid were used for comparison purposes. The results are presented in Table 2.

The DPPH assay revealed a moderate (relative to ascorbic acid) antioxidant capacity of all of the isolated lignan xylosides close to that of secoisolaricresinol: F2 > F1 > F4 > F3. The best result was observed for ssioriside (**2**), the IC_50_ value of which was ~2.5 and 2 times greater than that of fraction E2 and secoisolariciresinol, respectively. The results of the FRAP assay revealed a different distribution of AOA between the selected fractions compared to DPPH: F4 > F2 > F3 > F1. The highest value was obtained for F4 (10.5 ± 0.4 mM Fe^2+^/g sample), which was close to that of secoisolariciresinol and ~2 times greater than that of E2 (5.63 ± 0.23 mM Fe^2+^/g sample).

Since the existing protocols for AOA determination may vary significantly, a straightforward comparison of the obtained results and literature data may be difficult. However, in general, the AOA values measured in this study were consistent with those presented in other publications. Lee and co-authors [39] also tested the antioxidant properties of isolated ssioriside and lyoniside against DPPH radicals and came to the same conclusion that these compounds showed moderate activity (IC_50_ values of 60 and 112 µM, respectively), comparable to ascorbic acid (IC_50_ = 36.9 µM) as a control sample. In addition, both secoisolariciresinol and its diglucoside exhibited strong activity against the stable free radical DPPH [41]. However, in the case of FRAP, glycosides demonstrate weaker activity than aglycones, as described by Polat Kose and Gulcin [42].

## 4. Conclusions

The stems of *Rhododendron tomentosum* have been recognized as a new source for eight of lignan glyco- and xylosides (predominantly). This has been achieved through the use of targeted 2D nuclear magnetic resonance (NMR) and high-performance liquid chromatography-electrospray ionization-high resolution mass spectrometry (HPLC-ESI-HRMS).

Fractionation of the extract using reversed-phase high-performance liquid chromatography (HPLC) made it possible to obtain three lignans with a purity of more than 90%. In addition, a fraction consisting of two lignans was obtained with a purity of 98%. The main components were lyoniside (~0.37% of the weight of the vegetable raw materials) and ssioriside (~0.21% of the weight of vegetable raw materials). These are representatives of the aryltetraline and dibenzylbutane classes, respectively.

Studies using the DPPH and FRAP methods have shown that isolated glycosylated lignans exhibit moderate antioxidant capacity, comparable to that of secoisolariciresinol, and approximately 2 times greater than the antioxidant activity of the extract.

## Figures and Tables

**Table 1:** Tentative identification of lignans in the polyphenolic fraction of the extract by HPLC-ESI(–)-MS/MS.

No	Target Fragment	t_R_ *, min	*m*/*z* [M-H]^−^	Δ*m*/*z*, ppm	Elemental Composition	Putative Assignment
*Secoisolariciresinol and similar type structures*						
1	[C_20_H_25_O_6_]^−^	9.07	523.2198	2.26	C_26_H_36_O_11_	Secoisolariciresinol glucoside
2		10.96	539.2144 [M-H+FA]	1.89	C_25_H_34_O_10_	Secoisolariciresinol xyloside
3	[C_22_H_29_O_8_]^−^	9.41	583.2412	2.84	C_28_H_40_O_13_	5,5′-Dimethoxy-secoisolariciresinol glucoside
4		10.68	599.2353 [M-H+FA]	1.47	C_27_H_38_O_12_	Ssioriside
5		11.13	613.2510 [M-H+FA]	1.40	C_28_H_40_O_12_	5,5′-Dimethoxy-secoisolariciresinol rhamnoside (Chaenomiside F)
6	[C_21_H_27_O_7_]^−^	10.78	569.2252 [M-H+FA]	2.10	C_26_H_36_O_11_	Methoxysecoisolariciresinol xyloside
*5,5′-dimethoxy-isolariciresinol-type structures*						
7	[C_22_H_27_O_8_]^−^	9.49	597.2199 [M-H+FA]	1.42	C_27_H_36_O_12_	Lyoniside
*Other*						
8	-	11.73	597.2199 [M-H+FA]	2.03	C_27_H_36_O_12_	Not identified

* Retention time.

**Table 2:** Antioxidant activity in the polyphenolic fraction of the extract and lignan fractions isolated from the stems of *Rhododendron tomentosum* measured by two antioxidant assays (DPPH and FRAP). Results are expressed as mean ± standard deviation (*p* < 0.05, *n* = 3).

Sample	DPPH (0.3 mM) IC_50_, mg L^−1^	FRAP (4 min), mM Eq. Fe^2+^/g Sample
Fraction E2 (MeOH:H_2_O, 1:1, *v*/*v*)	37 ± 0	5.63 ± 0.23
Lyoniside (**7**)	21 ± 8	5.90 ± 0.42
Ssioriside (**4**)	15 ± 1	7.44 ± 0.34
5-Methoxysecoisolariciresinol 9-xyloside (**6**)	32 ± 2	6.42 ± 0.26
Secoisolariciresinol 9-xyloside (**2**) Prupaside (**8**)	27 ± 1	10.5 ± 0.4
Secoisolariciresinol *	29 ± 8	10.7 ± 0.1
Ascorbic acid *	10 ± 2	-

* Positive control.

## Data Availability

The data presented in this study are available in the article and Supplementary Materials.

## Supplementary Materials

The following supporting information can be downloaded at: https://www.mdpi.com/article/10.3390/antiox14040447/s1, Figure S1: ^1^H-^13^C HSQC NMR spectrum of the polyphenolic fraction; Figure S2: The carbon skeleton of two types of lignans identified from 2D NMR spectra; Figure S3: An example of the 2D-NMR workflow for unknown compound structure elucidation in a complex mixture; Figure S4: An example of the 2D-NMR workflow for unknown compound structure elucidation in a complex mixture; Figure S5: Preparative LC-PDA (280 nm) chromatogram of *Rhododendron tomentosum* stems extract; Figure S6: Tandem mass spectra of lyoniside; Figure S7: Tandem mass spectra of prupaside; Figure S8: Tandem mass spectra of secoisolariciresinol glucoside; Figure S9: Tandem mass spectra of secoisolariciresinol xyloside; Figure S10: Tandem mass spectra of methoxysecoisolariciresinol xyloside; Figure S11: Tandem mass spectra of 5,5-dimethoxy-secoisolariciresinol glucoside; Figure S12: Tandem mass spectra of ssioriside; Figure S13: Tandem mass spectra of 5,5-dimethoxy-seco-isolariciresinol rhamnoside; Figure S14: ^1^H NMR spectrum of F1; Figure S15: ^1^H-^13^C HSQC NMR spectrum of F1; Figure S16: ^1^H-^13^C HMBC NMR spectrum of F1; Figure S17: ^1^H NMR spectrum of F2; Figure S18: ^1^H-^13^C HSQC NMR spectrum of F2; Figure S19: ^1^H-^13^C HMBC NMR spectrum of F2; Figure S20: ^1^H NMR spectrum of F3; Figure S21: ^1^H-^13^C HSQC NMR spectrum of F3; Figure S22: ^1^H-^13^C HMBC NMR spectrum of F3; Figure S23: ^1^H-^1^H TOCSY spectrum of F3; Figure S24: ^1^H NMR spectrum of F4; Figure S25: ^1^H-^13^C HSQC NMR spectrum of F4; Figure S26: ^1^H-^13^C HMBC NMR spectrum of F4; Figure S27: ^1^H-^1^H TOCSY spectrum of F4; Figure S28: ^1^H–^1^H TOCSY and ^13^C–^1^H HMBC correlations of compounds 6 and 8. Table S1: ^1^H NMR spectroscopic data; Table S2: ^13^C NMR spectroscopic data.

## Abbreviations

2D NMR	Two-dimensional nuclear magnetic resonance
DPPH	2,2-diphenyl-1-picrylhydrazyl radical
FRAP	Ferric reducing antioxidant power
GC	Gas chromatography
HMBC	Heteronuclear multiple bond correlation
HPLC	High-performance liquid chromatography
HSQC	Heteronuclear single quantum coherence
MS	Mass spectrometry
RM	Raw materials
TOCSY	Total correlation spectroscopy
