# Supplementary material for: Bioactive Lignan Glycosides in Stems of Marsh Rosemary (Rhododendron tomentosum): Non-Targeted Screening and Identification Using Two-Stage Analytical Strategy
Source: Antioxidants (Basel). 2025 Apr 8;14(4):447. doi: 10.3390/antiox14040447 (PMC12024211; doi:10.3390/antiox14040447)
Supplement: Supplementary file 1 [file antioxidants-14-00447-s001.zip › antioxidants-3562823-supplementary.pdf]

SUPPORTING INFORMATION

Bioactive Lignan Glycosides in Stems of Marsh Rosemary (*Rhododendron tomentosum*): Non-Targeted Screening and Identification Using Two-Stage Analytical Strategy

Anna V. Faleva *, Danil I. Falev, Aleksandra A. Onuchina, Nikolay V. Ulyanovskii and Dmitry S. Kosyakov

Laboratory of Natural Compounds Chemistry and Bioanalytics, Core Facility Center "Arktika", M. V. Lomonosov Northern (Arctic) Federal University, Northern Dvina Emb. 17, 163002 Arkhangelsk, Russia

* Correspondence: a.bezumova@narfu.ru

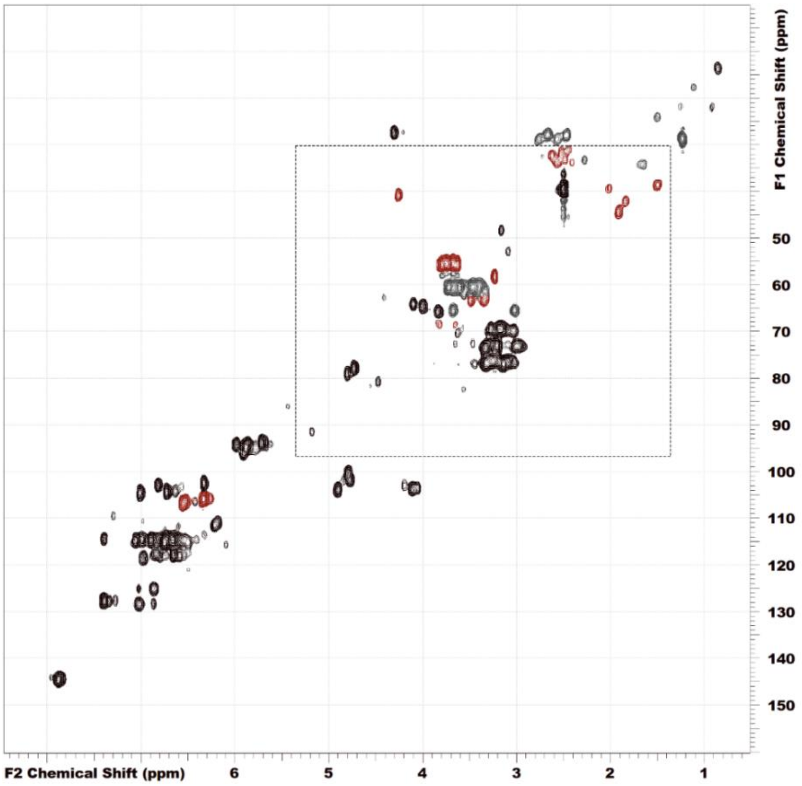

Figure S1. ^{1}H-^{13}C HSQC NMR spectrum of the polyphenolic fraction of the extract isolated from stems of *Rhododendron tomentosum*. The lignan signals are highlighted in red

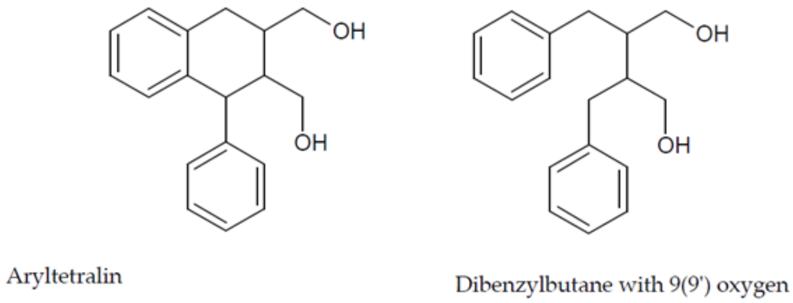

Figure S2. The carbon skeleton of two types of lignans (aryltetralin and dibenzylbutane structures with 9(9') oxygen) has been identified from 2D NMR spectra

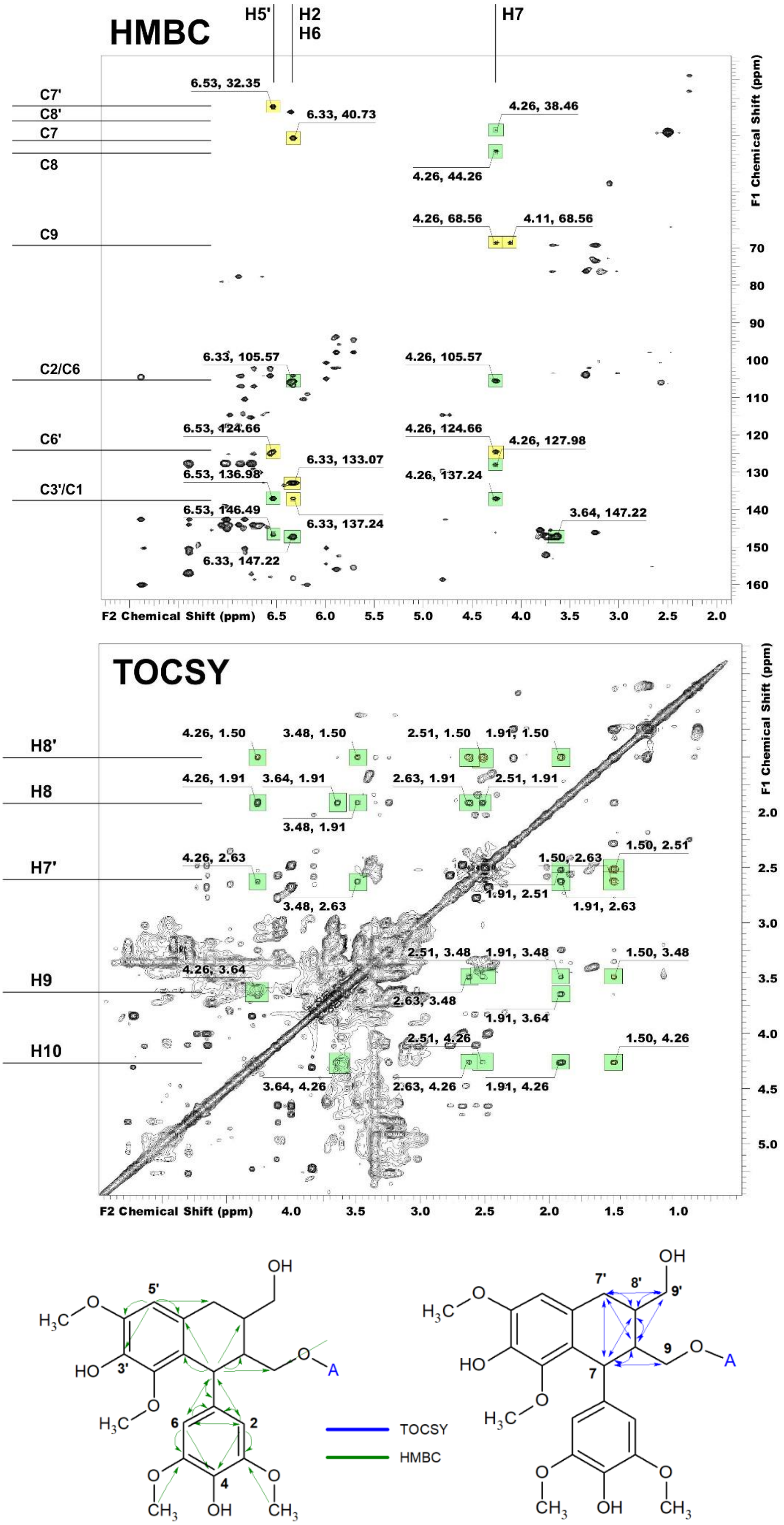

Figure S3. An example of 2D-NMR workflow for unknown compound structure elucidation in complex mixture; the signals corresponding to the structure of 5,5'-dimethoxy-isolariciresinol are shown

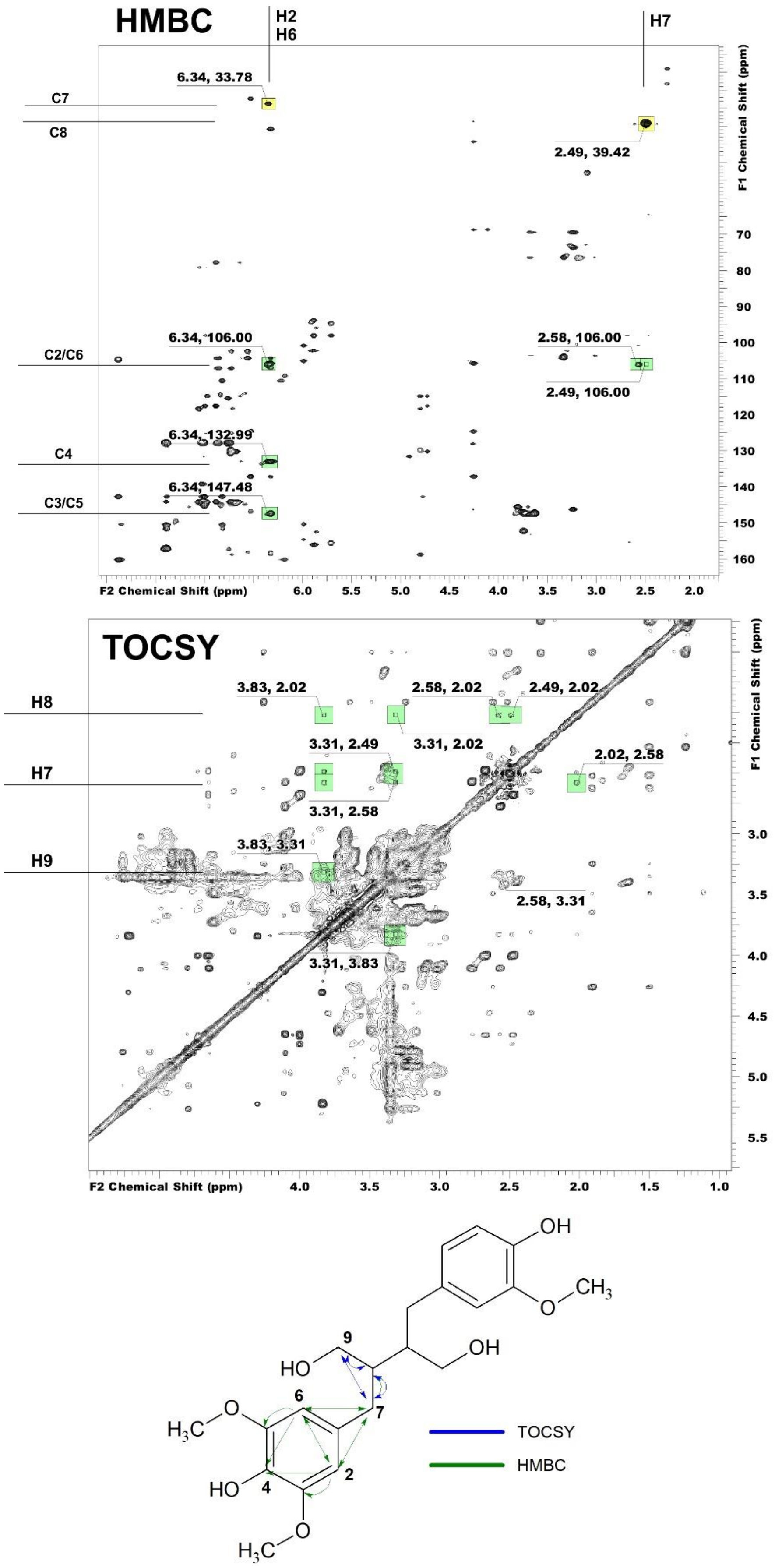

Figure S4. An example of 2D-NMR workflow for unknown compound structure elucidation in complex mixture; the signals corresponding to the structure of secoisolaricresinol are shown

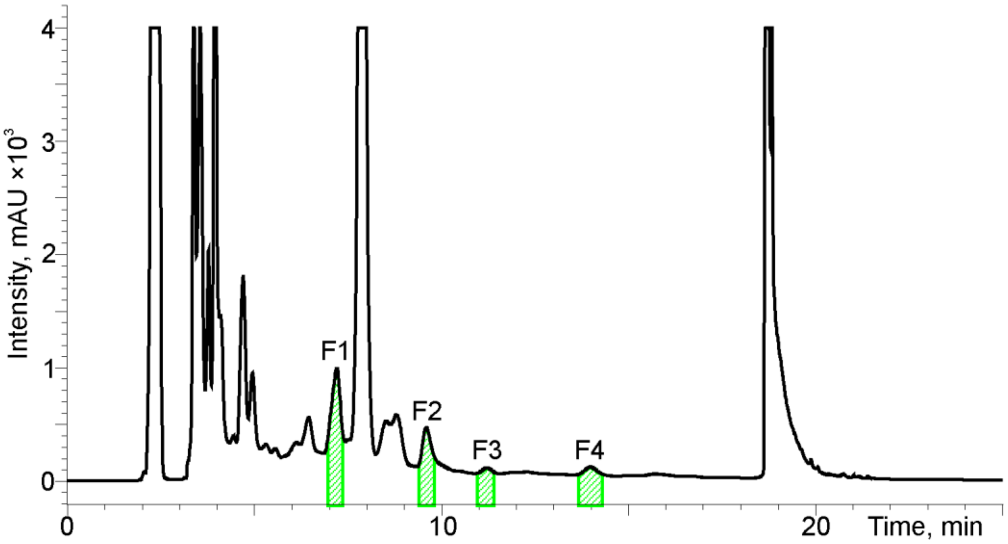

Figure S5. Preparative LC-PDA (280 nm) chromatogram of *Rhododendron tomentosum* stems extract (15 mg/mL) and the fraction collection periods (green).

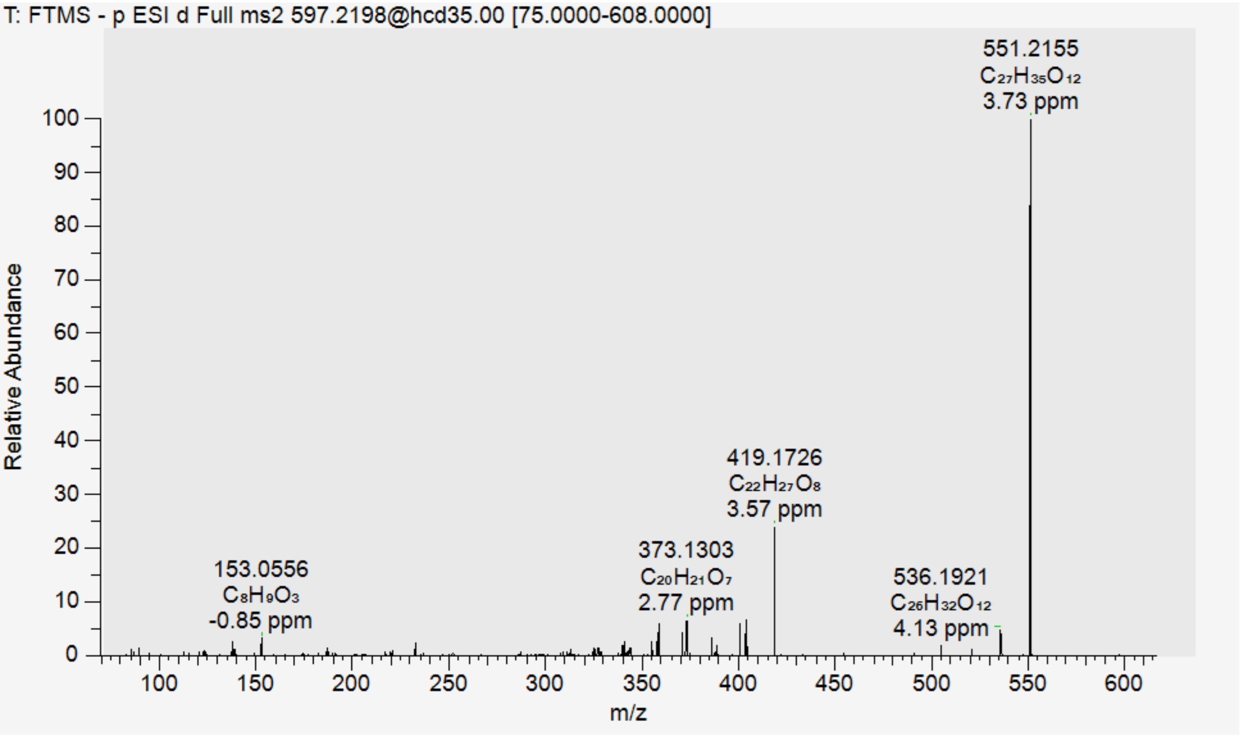

Figure S6. Tandem mass spectra of lyoniside (precursor ion 597.2199) observed in negative ion detection mode

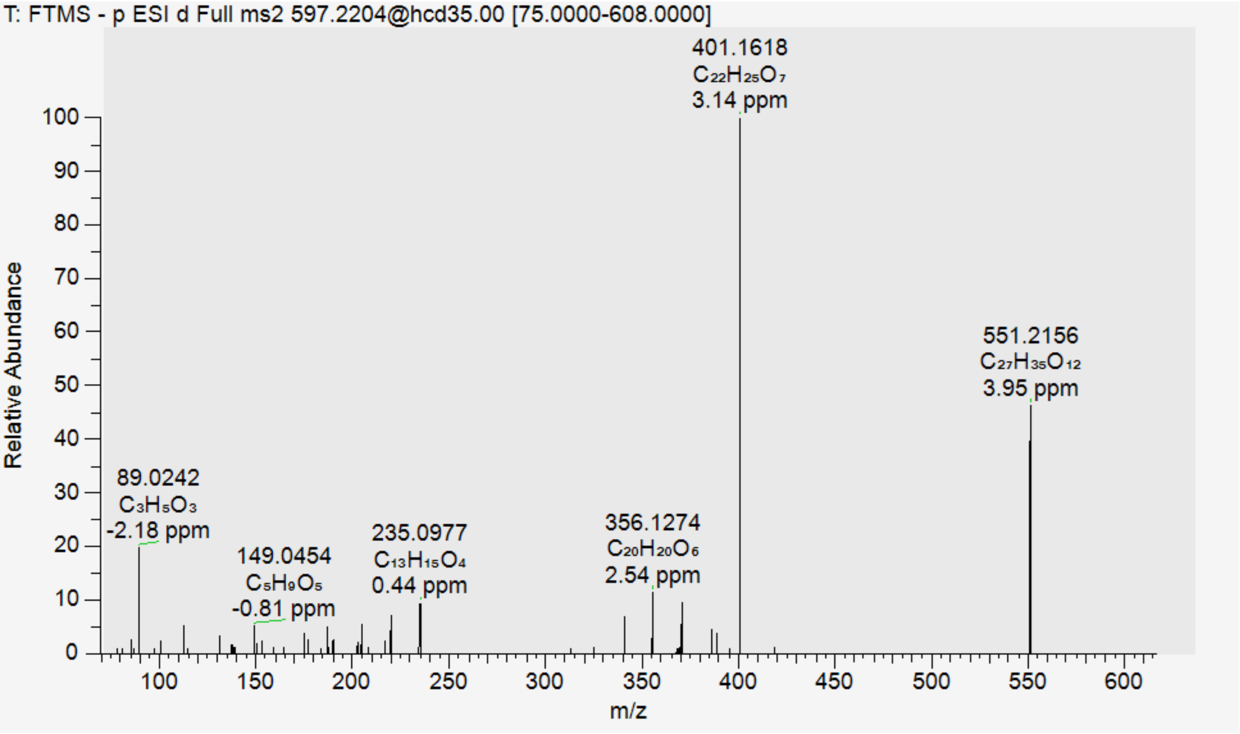

Figure S7. Tandem mass spectra of prupaside (precursor ion 597.2199) observed in negative ion detection mode

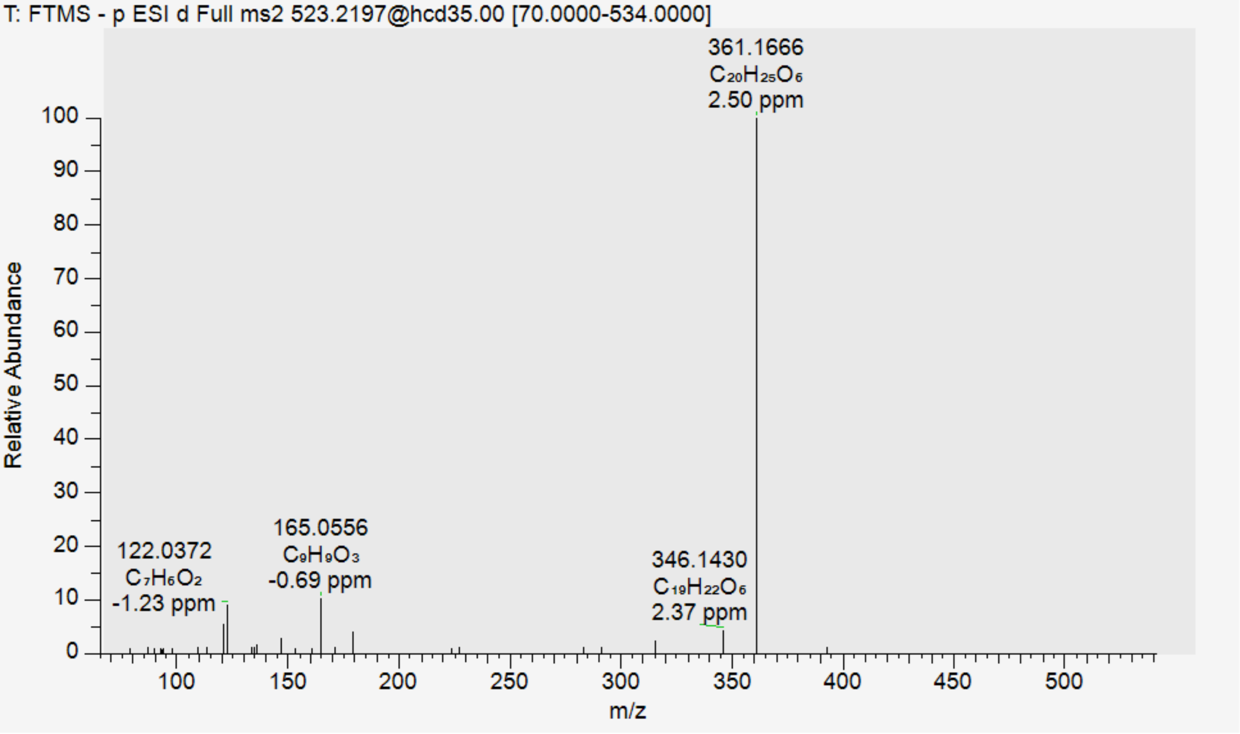

Figure S8. Tandem mass spectra of secoisolariciresinol glucoside (precursor ion 523.2198) observed in negative ion detection mode

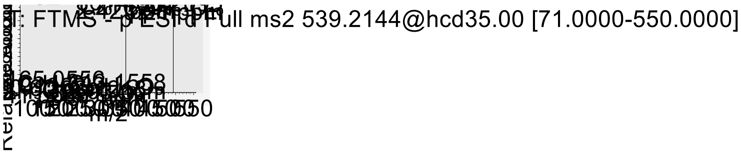

Figure S9. Tandem mass spectra of secoisolariciresinol xyloside (precursor ion 539.2144) observed in negative ion detection mode

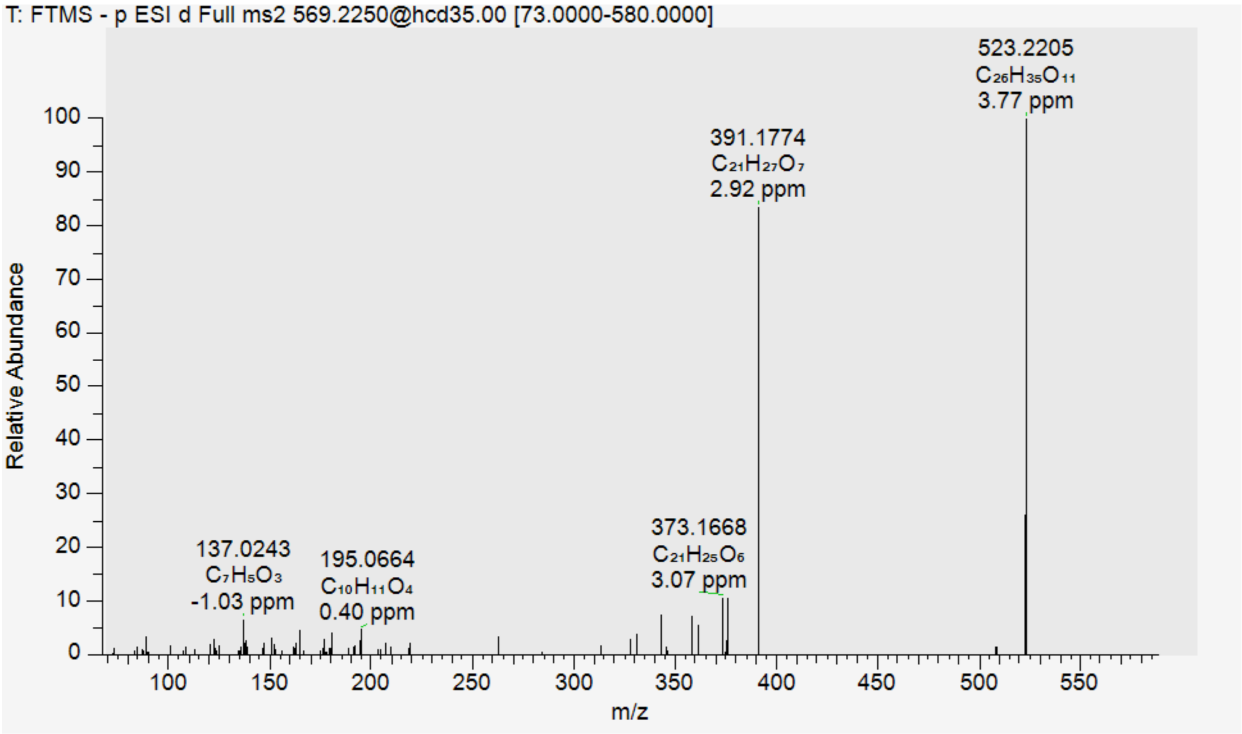

Figure S10. Tandem mass spectra of methoxysecoisolariciresinol xyloside (precursor ion 569.2252) observed in negative ion detection mode

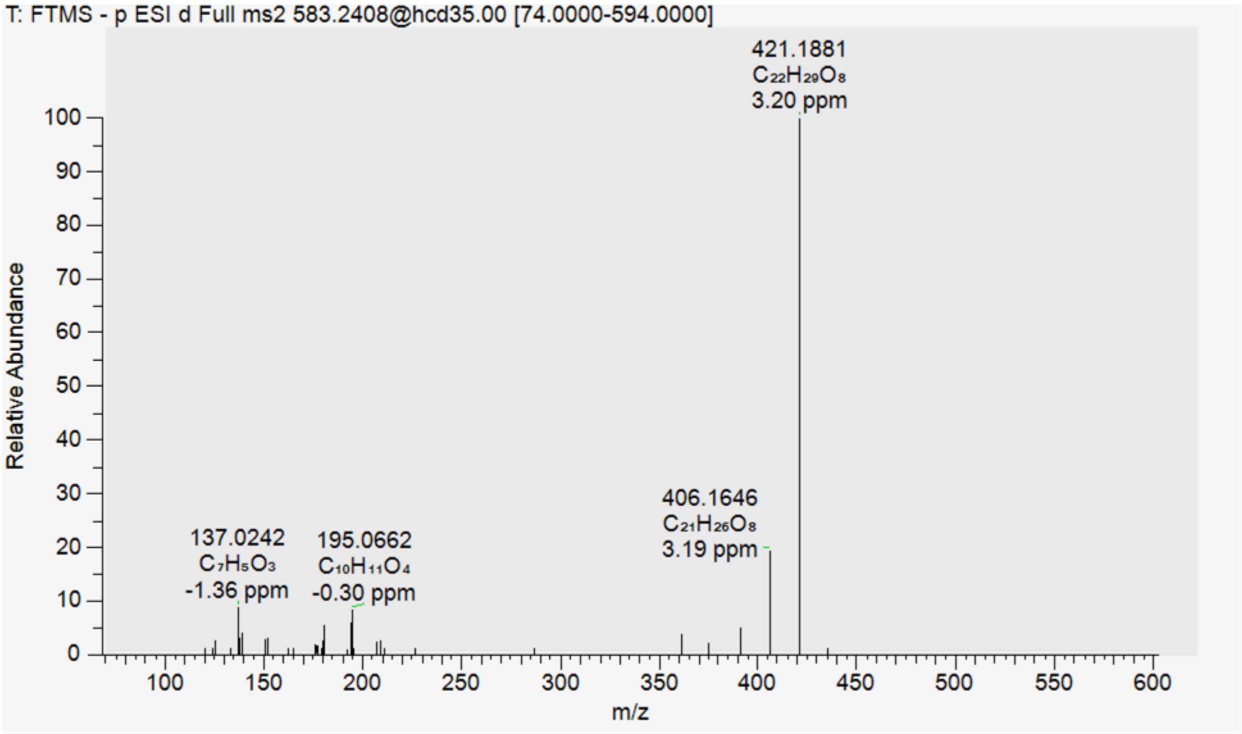

Figure S11. Tandem mass spectra of 5,5-dimethoxy-secoisolariciresinol glucoside (precursor ion 583.2412) observed in negative ion detection mode

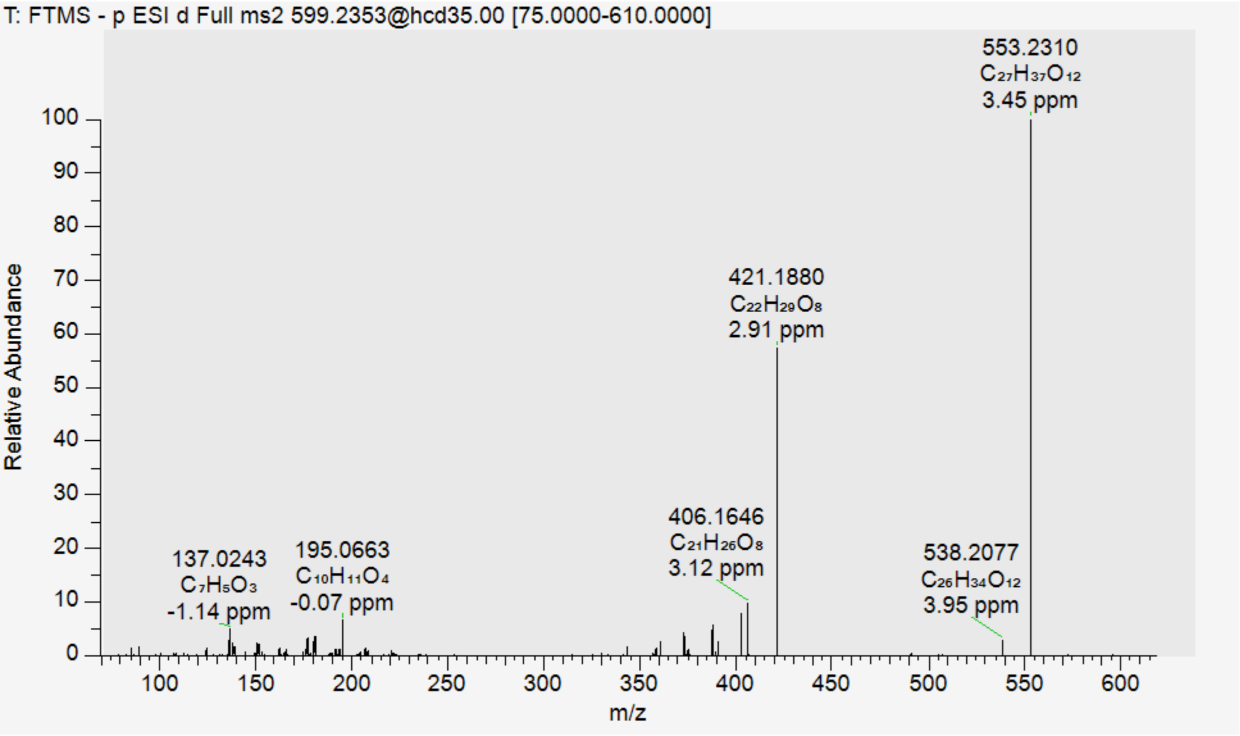

Figure S12. Tandem mass spectra of ssioriside (precursor ion 599.2353) observed in negative ion detection mode

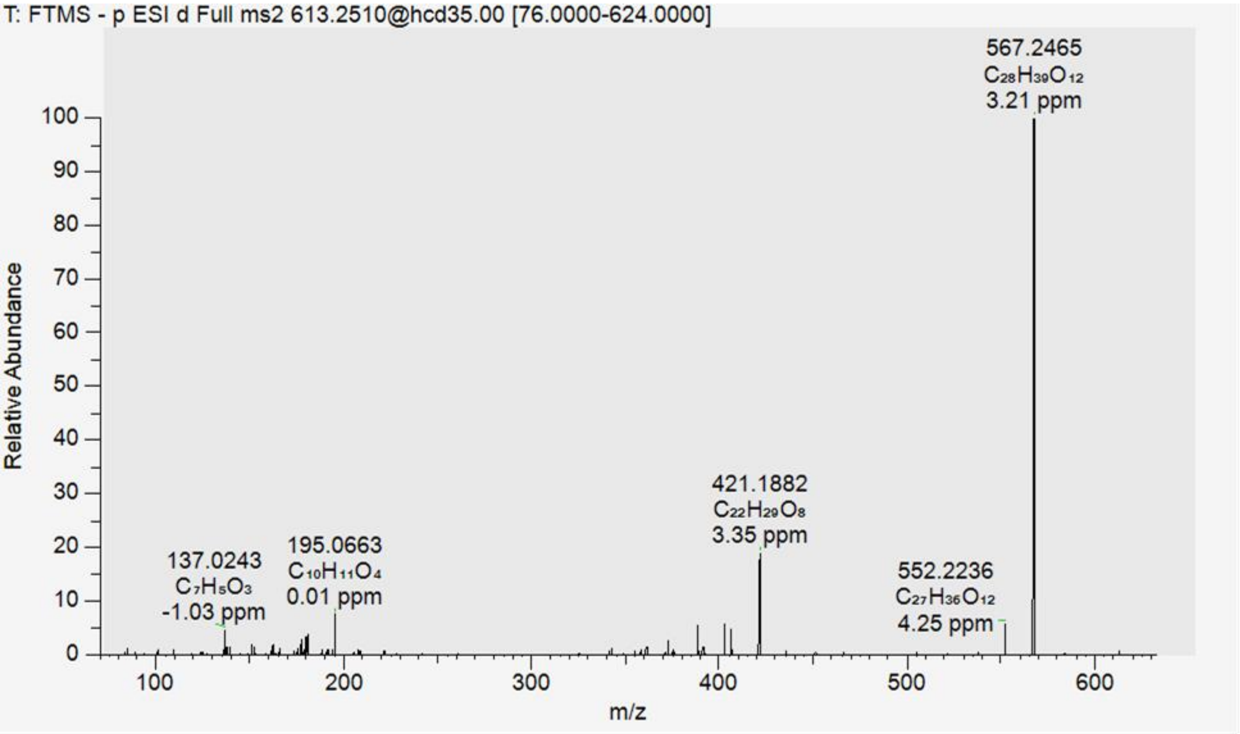

Figure S13. Tandem mass spectra of 5,5-dimethoxy-seco-isolariciresinol rhamnoside (precursor ion 613.2510) observed in negative ion detection mode

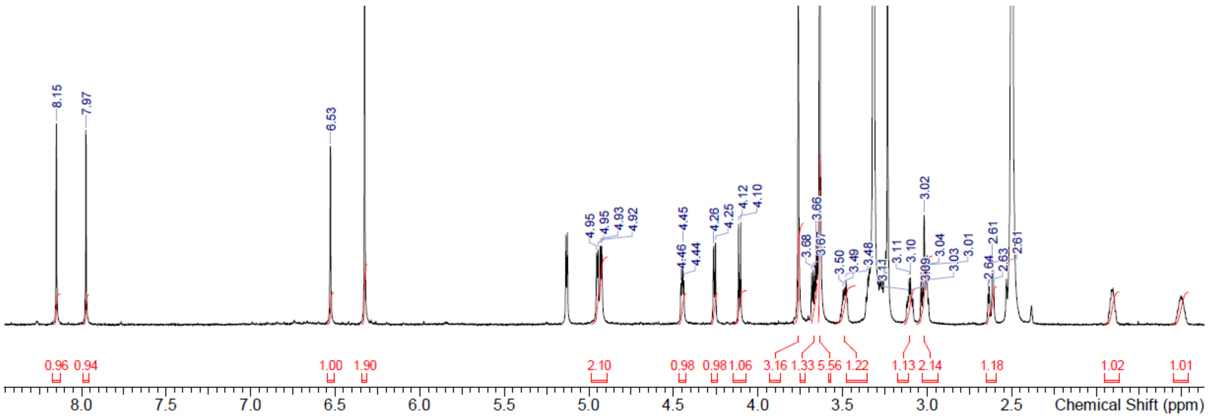

Figure S14. ^{1}H NMR spectrum of F1

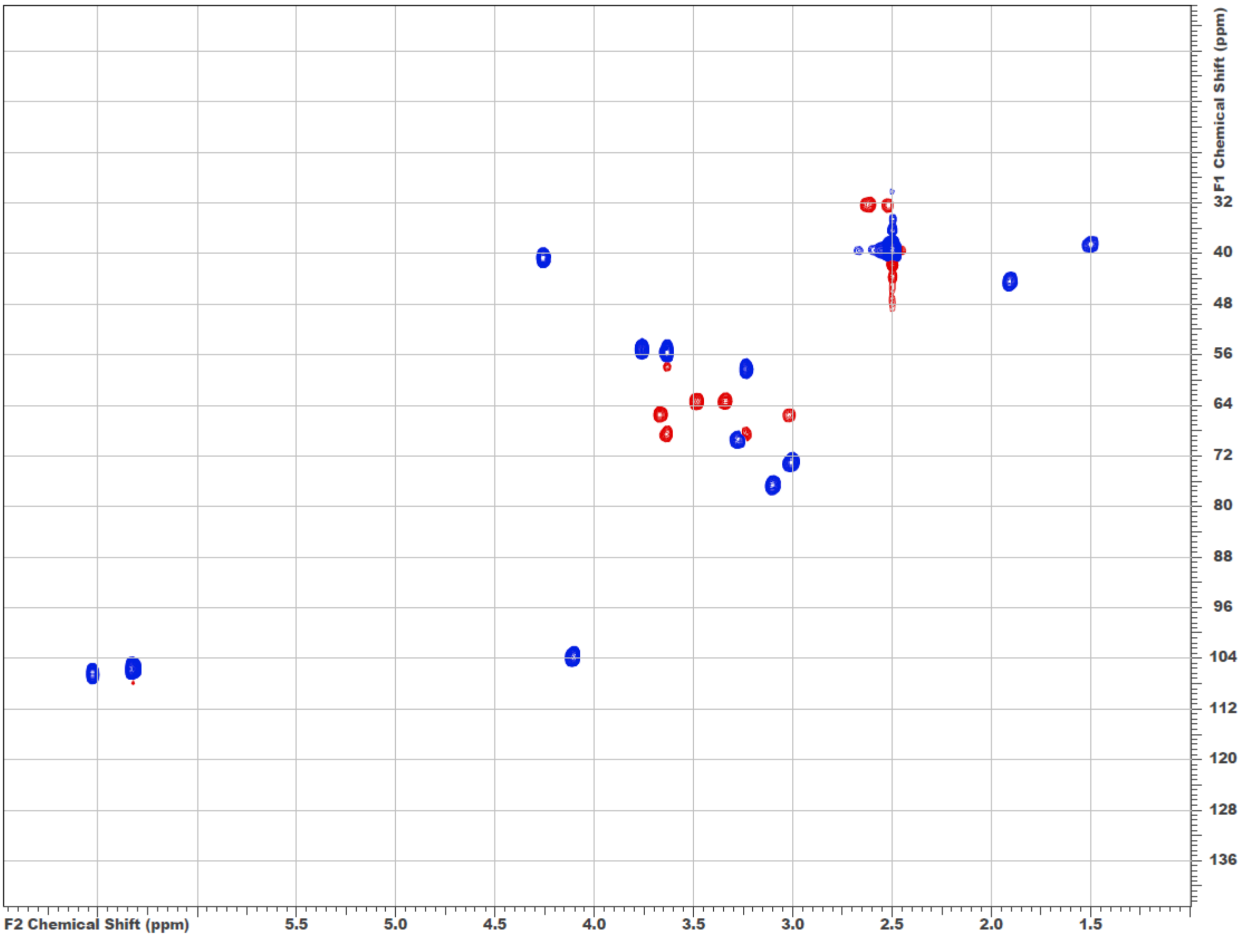

Figure S15. ^{1}H-^{13}C HSQC NMR spectrum of F1

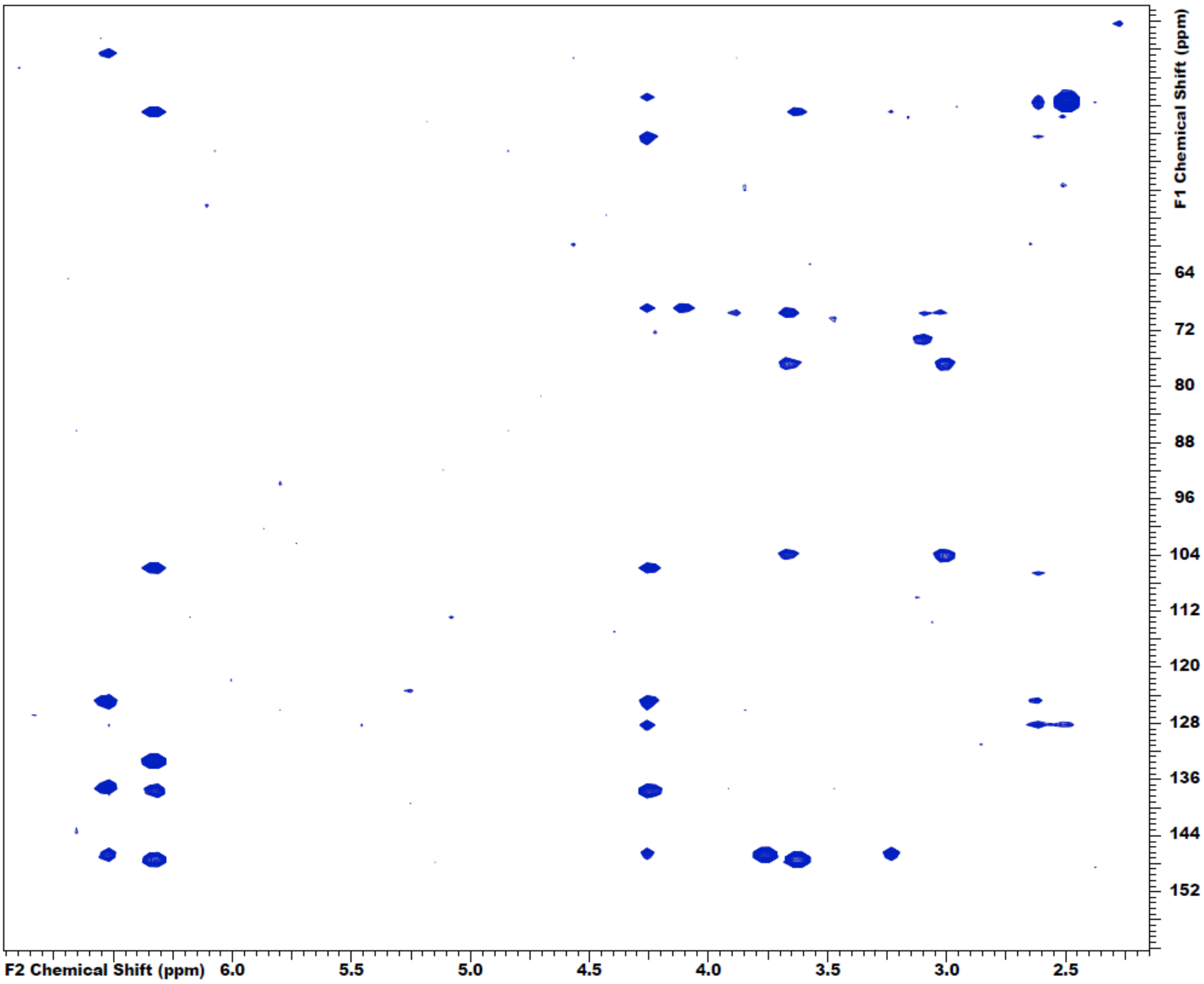

Figure S16. ^{1}H-^{13}C HMBC NMR spectrum of F1

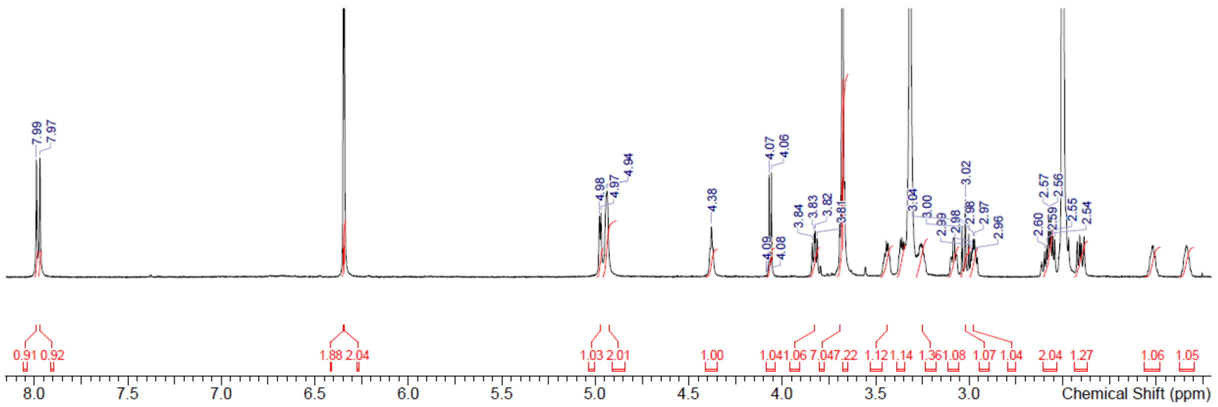

Figure S17. ^{1}H NMR spectrum of F2

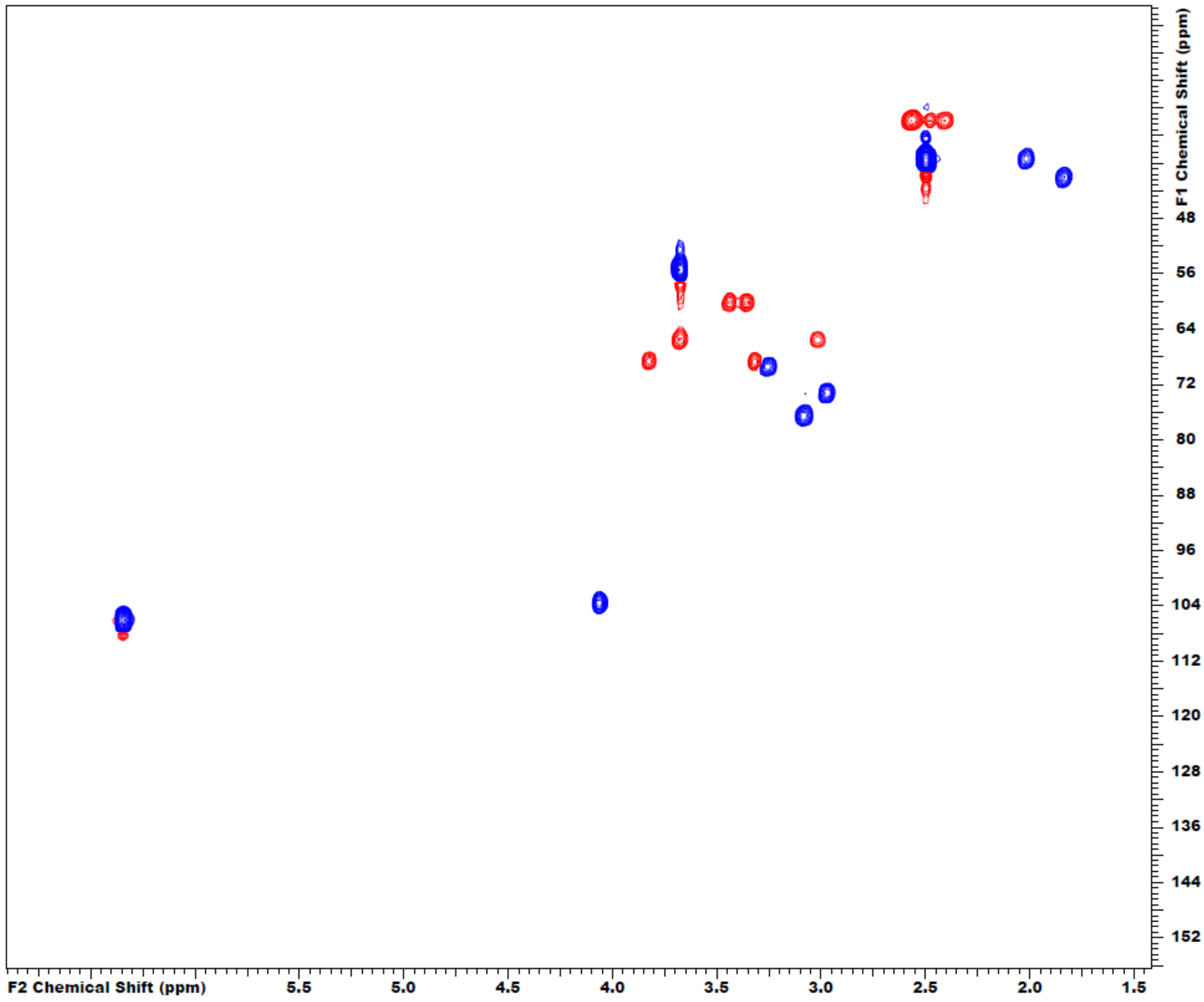

Figure S18. ^{1}H-^{13}C HSQC NMR spectrum of F2

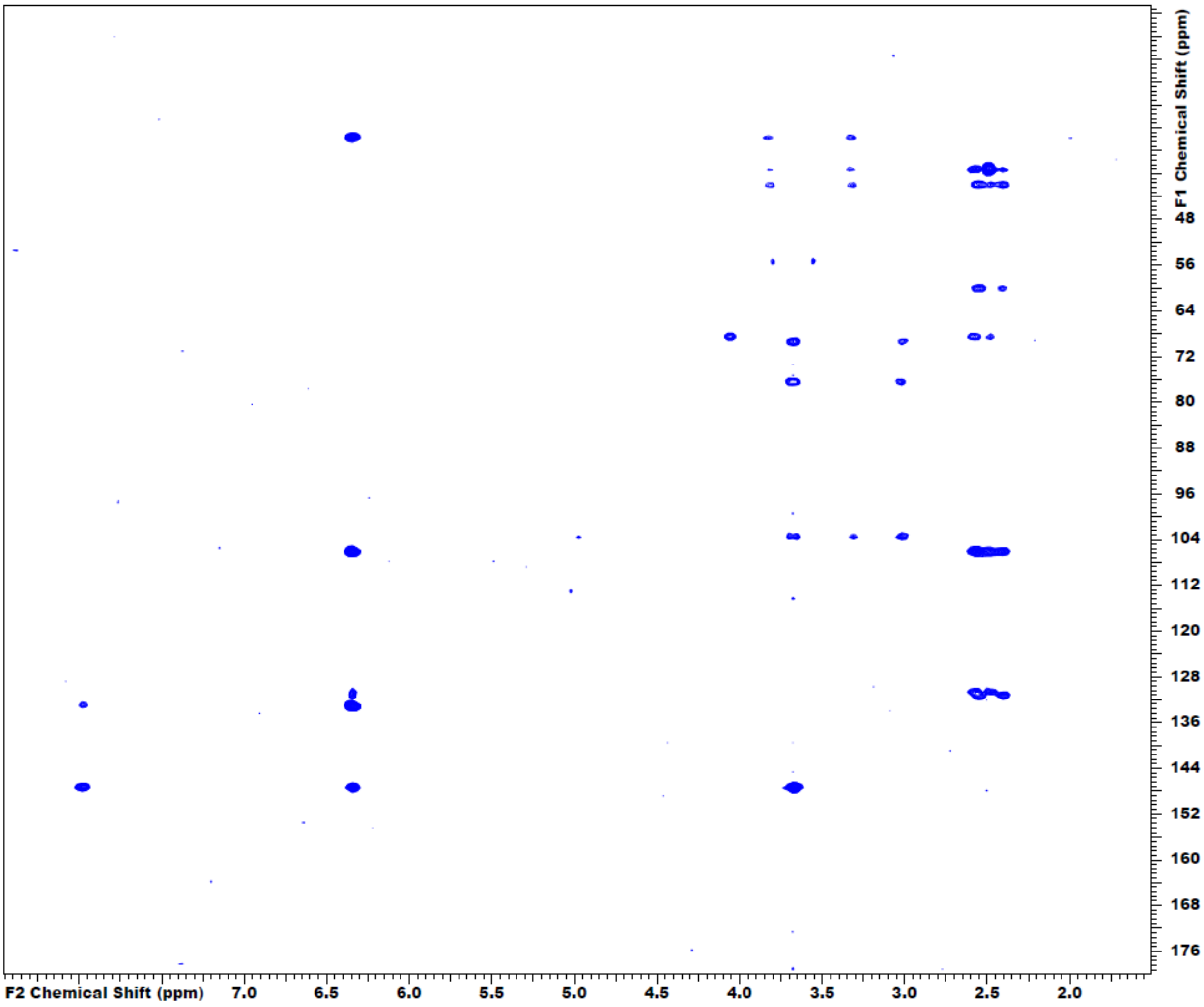

Figure S19. ^{1}H-^{13}C HMBC NMR spectrum of F2

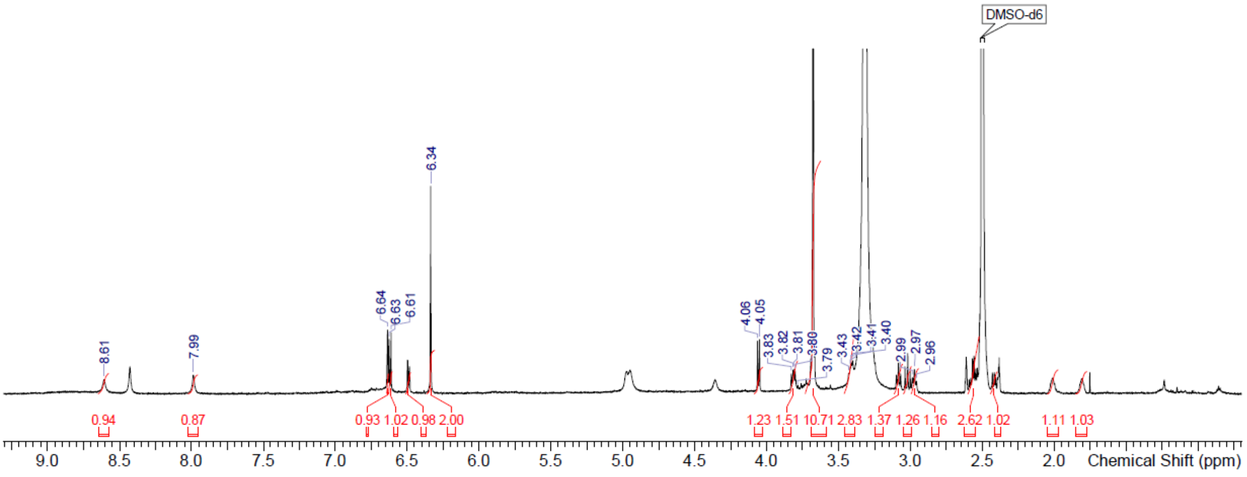

Figure S20. ^{1}H NMR spectrum of F3

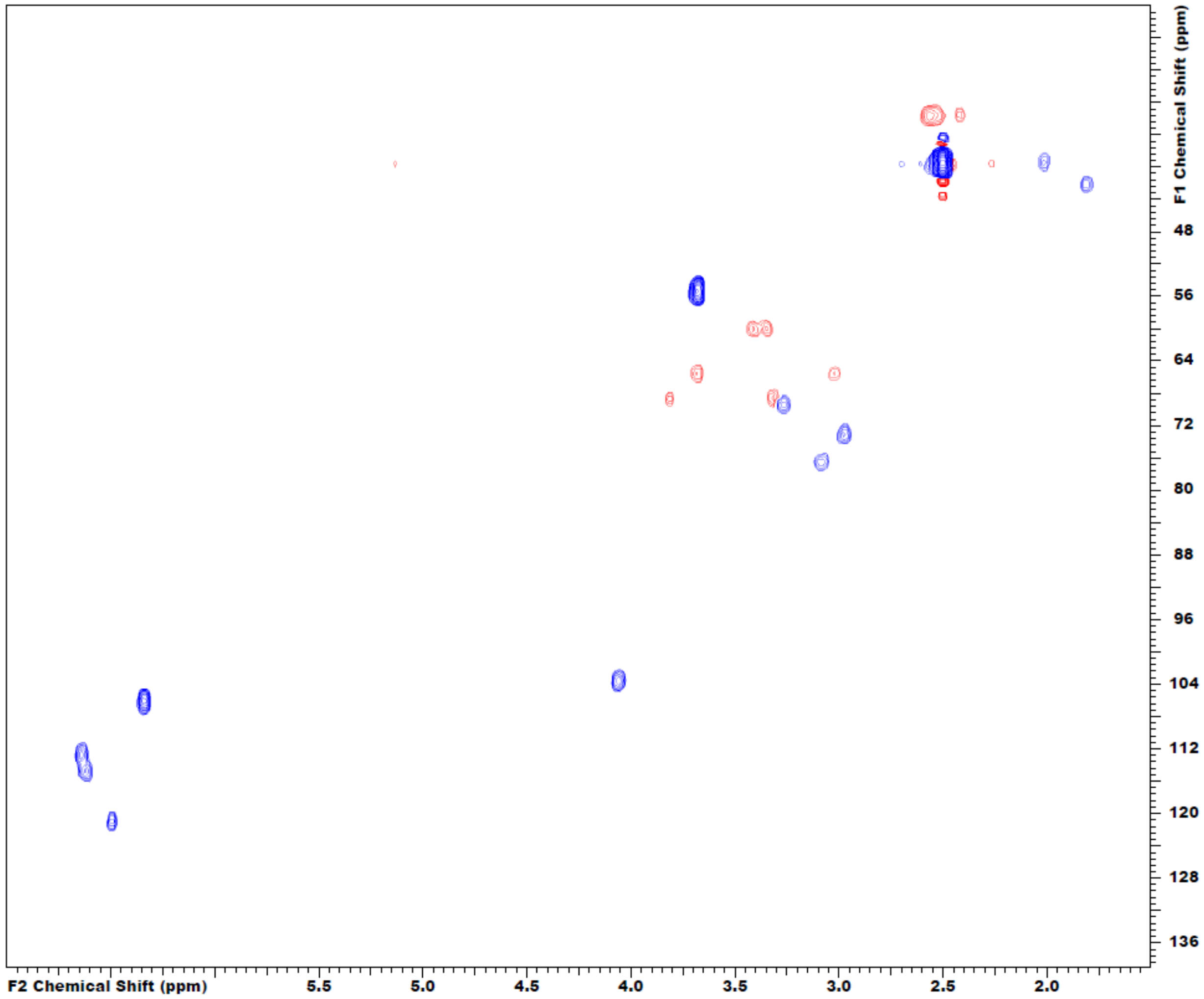

Figure S21. ^{1}H-^{13}C HSQC NMR spectrum of F3

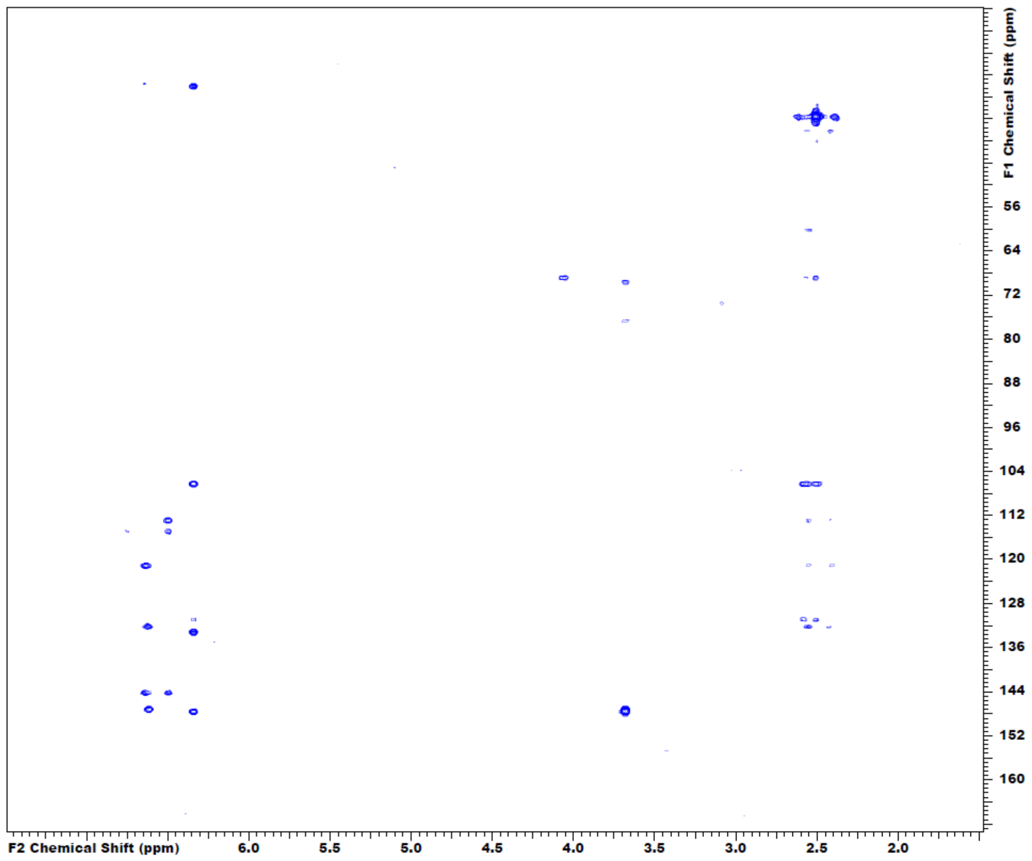

Figure S22. ^{1}H-^{13}C HMBC NMR spectrum of F3

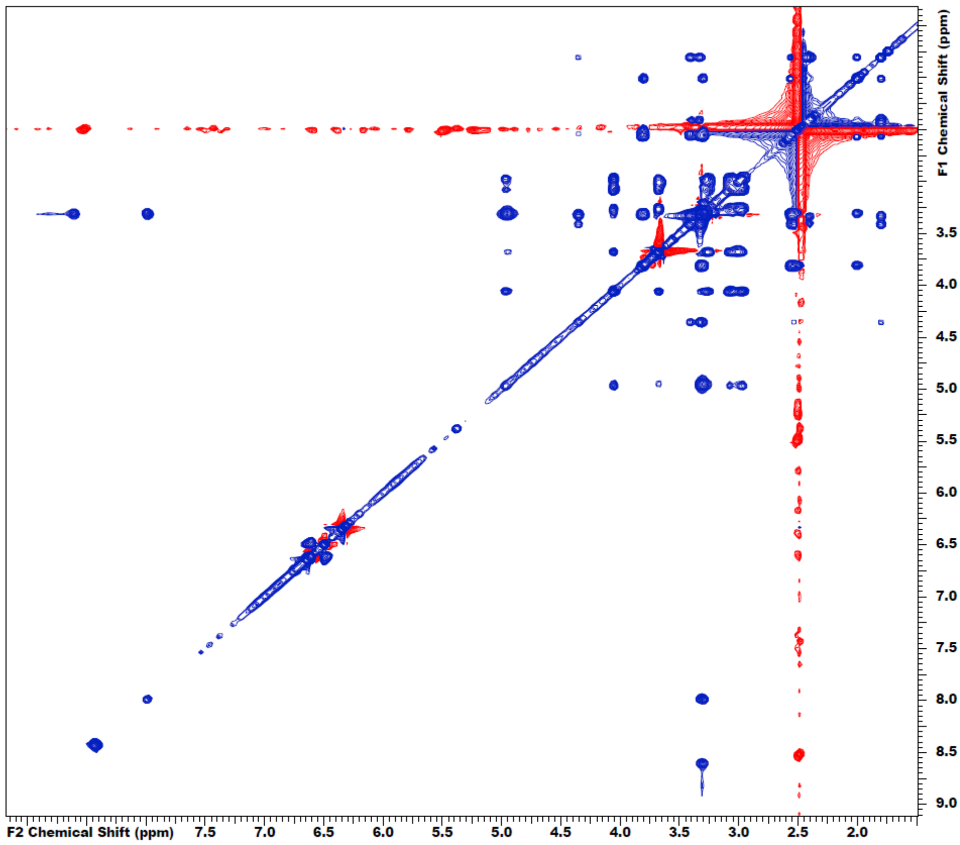

Figure S23. ^{1}H-^{1}H TOCSY spectrum of F3

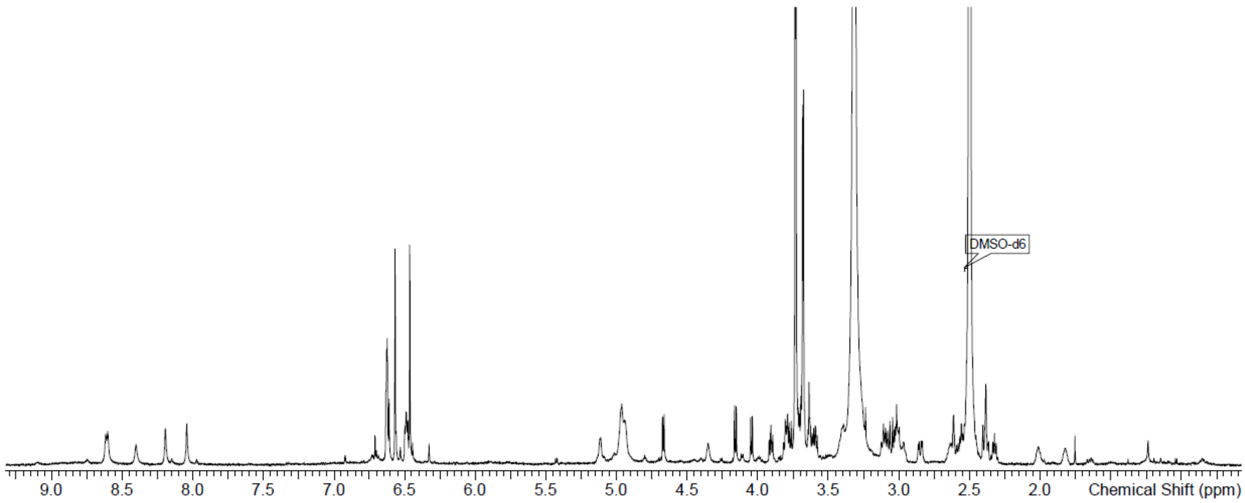

Figure S24. ^{1}H NMR spectrum of F4

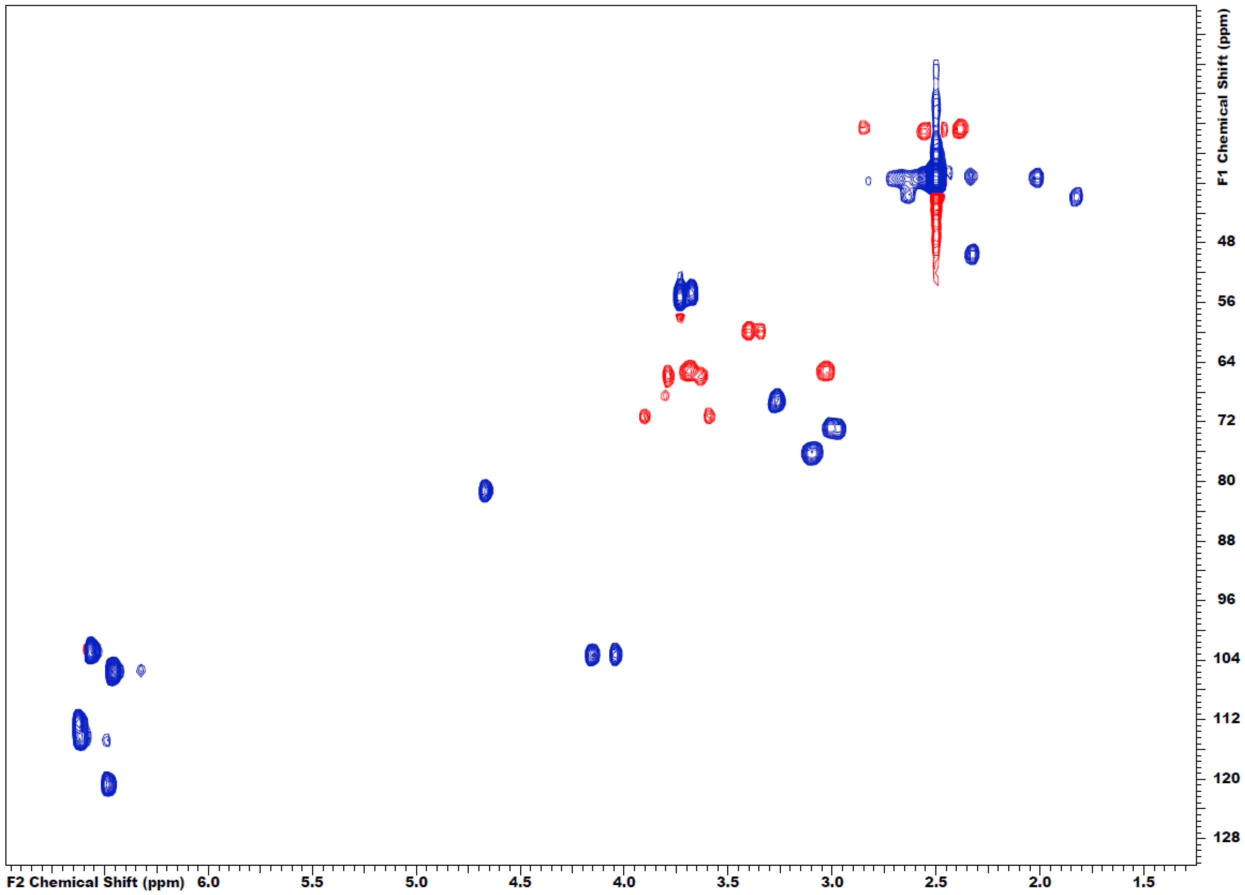

Figure S25. ^{1}H-^{13}C HSQC NMR spectrum of F4

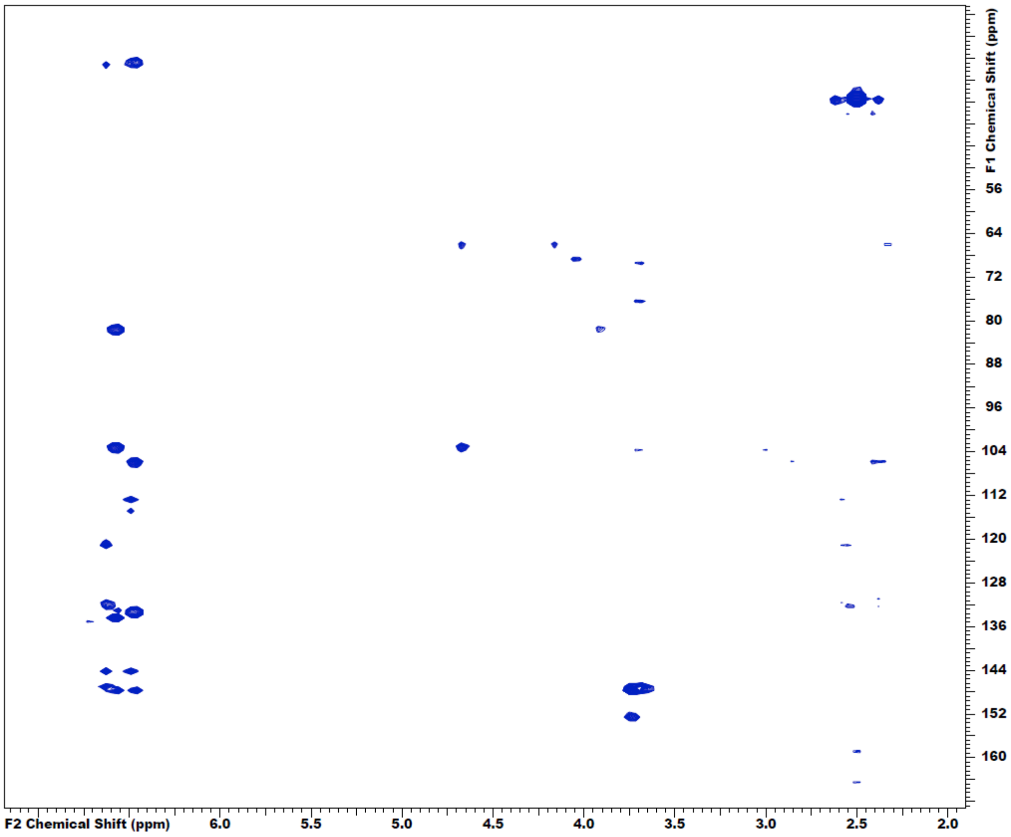

Figure S26. ^{1}H-^{13}C HMBC NMR spectrum of F4

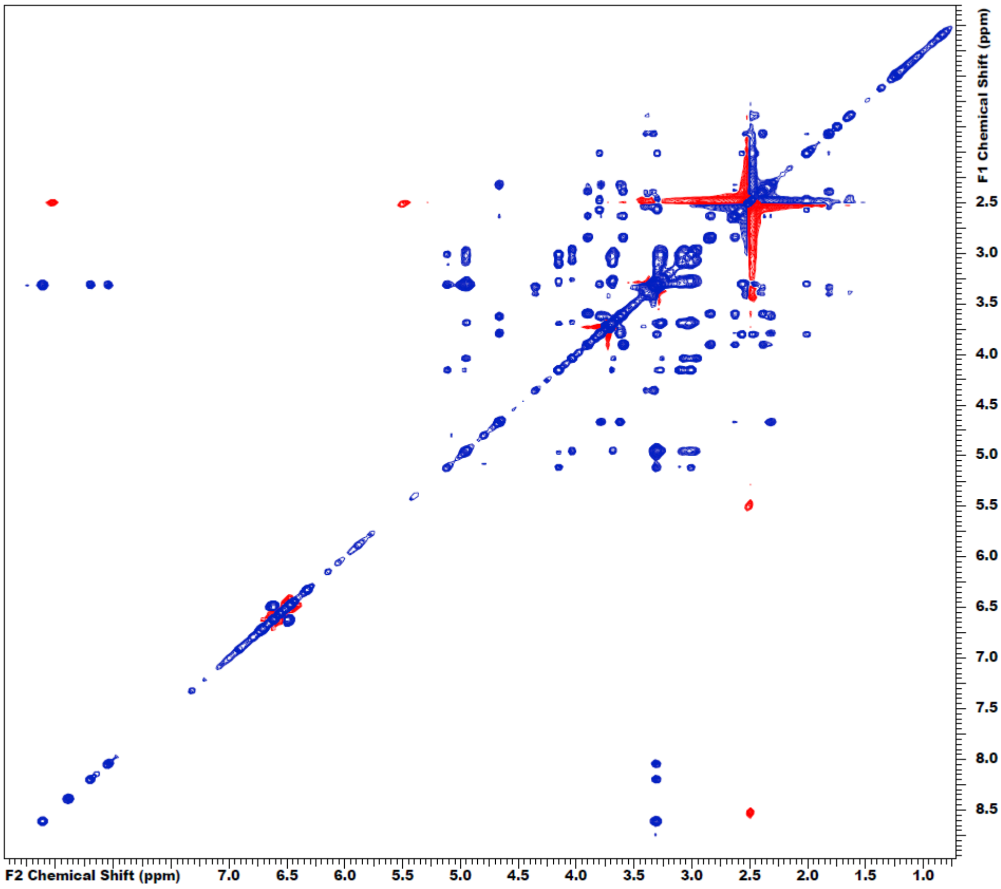

Figure S27. ^{1}H-^{1}H TOCSY spectrum of F4

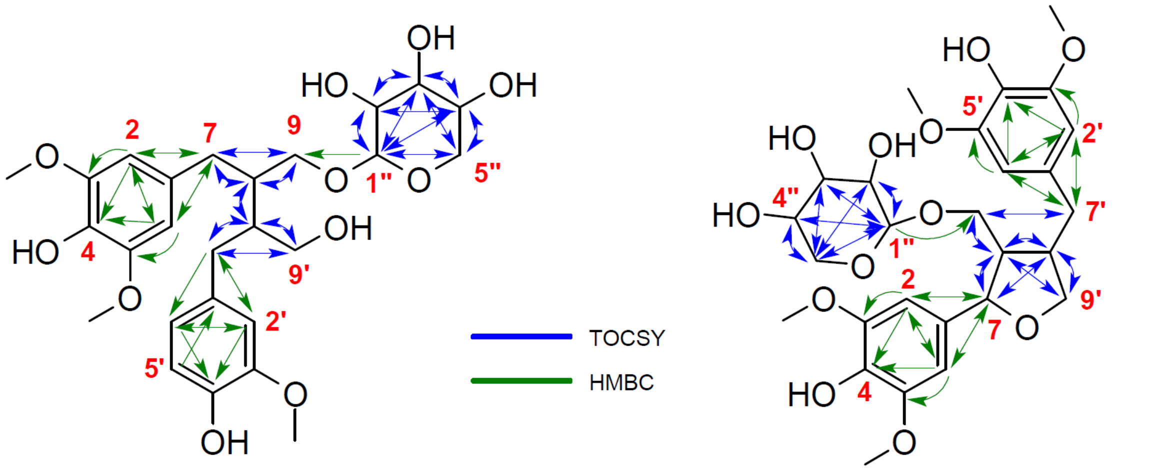

5-Methoxysecoisolariciresinol xyloside **Prupaside**

Figure S28. The ${}^{1}H$–${}^{1}H$ TOCSY and ${}^{13}C$–${}^{1}H$ HMBC correlations of compound **6** and **8.** Explanation for different colors: red – the atom numbers; blue – TOCSY correlations; green – HMBC correlations

Table S1. ^{1}H NMR Spectroscopic data (600 MHz, DMSO-d_6) for fractions F1–F4. The signals that can be used as diagnostic NMR signals distinguishing these lignans are highlighted in bold.

proton	F1<br>Lyoniside	F2<br>Ssioriside	F3<br>5-Methoxysecoisolariciresinol xyloside	F4<br>Secoisolariciresinol xyloside	 Prupaside
			δH, ppm (J, Hz)		
1	-	-	-	-	-
2	6.33 (s)	6.35 (s)	6.34 (s)	6.62 (m)	6.56 (s)
3	-	-	-	-	-
4	-	-	-	-	-
5	-	-	-	6.62 (m)	-
6	6.33 (s)	6.35 (s)	6.34 (s)	6.48 (m)	6.56 (s)
7	**4.26 (d, 6.6)**	**2.57 and 2.48 (overlapped)**	**2.54 (overlapped)**	**2.55 and 2.47 (overlapped)**	**4.67 (d, 7.15)**
8	**1.9 (m)**	**2.02 (m)**	**2.01 (m)**	**2.01 (m)**	**2.33 (q, 7.15)**
9	**3.64 and 3.23 (m)**	**3.83 and 3.32 (dd, 9.35 and 6.6)**	**3.81 and 3.31 (m)**	**3.80 and 3.31 (overlapped)**	**3.79 and 3.63**
1'	-	-	-	-	-
2'	-	6.35 (s)	6.63 (d, 1.65)	6.62 (m)	6.46 (s)
3'	-	-	-	-	-
4'	-	-	-	-	-
5'	6.53 (s)	-	6.62 (d, 8.25)	6.62 (m)	-
6'	-	6.35 (s)	6.49 (dd, 7.9 and 1.6)	6.48 (m)	6.46 (s)
7'	**2.61 and 2.52 (m)**	**2.57 and 2.41 (dd, 13.75 and 8.25)**	**2.58 and 2.42 (overlapped)**	**2.55 and 2.47 (overlapped)**	**2.85 and 2.39 (dd, 13.2 and 4.4)**
8'	**1.5 (m)**	**1.84 (m)**	**1.81 (m)**	**1.82 (m)**	**2.63 (overlapped)**
9'	**3.49 and 3.34 (m)**	**3.44 and 3.36 (overlapped)**	**3.41 and 3.34 (overlapped)**	**3.40 and 3.34 (overlapped)**	**3.90 and 3.59**
1''	4.11 (d, 7.7)	4.06 (d, 7.15)	4.06 (d, 7.7)	4.04 (d, 7.15)	4.16 (d, 7.7)
2''	3.01 (m)	2.97 (m)	2.97 (m)	2.97 (m)	3.01 (m)
3''	3.1 (m)	3.08 (m)	3.08 (t, 8.8)	3.1 (m)	3.1 (m)
4''	3.27 (m)	3.26 (m)	3.27 (m)	3.27 (m)	3.27 (m)
5''	3.67 and 3.01 (m)	3.68 and 3.02 (m; t, 10.7)	3.68 and 3.02 (m; t, 10.7)	3.69 and 3.03 (m)	3.69 and 3.03 (m)
OCH_3-3 (5)	3.64 (s)	3.68 (s)	3.68 (s)	3.68 (s)	3.73 (s)
OCH_3-2'	3.24 (s)	-	-	-	-
OCH_3-4'	3.76 (s)	-	-	-	-

Table S2. ^{13}C NMR Spectroscopic data (150 MHz, DMSO-d_6) for fractions F1–F4 (chemical shifts based on HSQC and HMBC correlation peaks). The signals that can be used as diagnostic NMR signals distinguishing these lignans are highlighted in bold.

carbon	F1<br>Lyoniside	F2<br>Ssioriside	F3<br>5-Methoxysecoisolariciresinol xyloside<br>δC, ppm	F4<br>Secoisolariciresinol xyloside	Prupaside
1	137.34	130.40	130.84	131.92	n/d
2	105.65	105.92	106.02	112.68	103.01
3	147.32	147.09	147.60	147.06	147.58
4	133.11	132.88	133.26	144.22	134.3
5	147.32	147.09	147.60	114.72	147.58
6	105.65	105.92	106.02	120.96	103.01
7	**40.75**	**33.60**	**33.56**	**33.24**	**81.5**
8	**44.35**	**39.25**	**39.49**	**39.51**	**49.73**
9	**68.63**	**68.52**	**68.68**	**68.44**	**66.06**
1'	124.59	131.00	132.13	131.92	n/d
2'	146.16	105.92	112.62	112.68	105.75
3'	137.00	147.09	147.17	147.06	147.58
4'	146.68	132.88	144.21	144.22	133.37
5'	106.41	147.09	114.72	114.72	147.58
6'	127.99	105.92	120.93	120.96	105.75
7'	**32.28**	**33.60**	**33.66**	**33.24**	**32.75**
8'	**38.65**	**41.94**	**42.10**	**41.99**	**41.89**
9'	**63.35**	**60.00**	**60.08**	**60.07**	**71.48**
1''	103.74	103.43	103.60	103.51	103.51
2''	73.00	73.11	73.08	73.18	73.04
3''	76.75	76.36	76.54	76.44	76.44
4''	69.38	69.38	69.37	69.38	69.38
5''	65.40	65.41	65.53	65.44	65.44
OCH_3-3 (5)	55.66	55.24	55.27	54.96	55.5
OCH_3-2'	58.19	-	-	-	-
OCH_3-4'	55.23	-	-	-	-
